# Modelling of compression ignition engine by soft computing techniques (ANFIS-NSGA-II and RSM) to enhance the performance characteristics for leachate blends with nano-additives

**DOI:** 10.1038/s41598-023-42353-1

**Published:** 2023-09-18

**Authors:** Osama Khan, Mohd Parvez, Pratibha Kumari, Ashok Kumar Yadav, Wasim Akram, Shadab Ahmad, Samia Parvez, Mohammad Javed Idrisi

**Affiliations:** 1 Department of Mechanical Engineering, Jamia Millia Islamia, New Delhi, 110025 India; 2 Department of Mechanical Engineering, Al Falah University, Faridabad, Haryana 121004 India; 3 Department of Mechanical Engineering, KIET Group of Institutions, Ghaziabad, UP 201206 India; 4 Department of Mechanical Engineering, Raj Kumar Goel Institute of Technology, Ghaziabad, UP 201003 India; 5 Department of Mechanical Engineering, Mewat Engineering College, Palla, Mewat, Harayana India; 6 Department of Civil Engineering, Jamia Millia Islamia, New Delhi, 110025 India; 7 Department of Mathematics, College of Natural and Computational Science, Mizan-Tepi University, Tepi, Ethiopia

**Keywords:** Computational models, Biological techniques, Energy science and technology

## Abstract

Integrating nanoparticles in waste oil-derived biodiesel can revolutionize its performance in internal combustion engines, making it a promising fuel for the future. Nanoparticles act as combustion catalysts, enhancing combustion efficiency, reducing emissions, and improving fuel economy. This study employed a comprehensive approach, incorporating both quantitative and qualitative analyses, to investigate the influence of selected input parameters on the performance and exhaust characteristics of biodiesel engines. The focus of this study is on the potential of using oils extracted from food waste that ended up in landfills. The study's results are analysed and compared with models created using intelligent hybrid prediction approaches including adaptive neuro-fuzzy inference system, Response surface methodology-Genetic algorithm, and Non sorting genetic algorithm. The analysis takes into account engine load, blend percentage, nano-additive concentration, and injection pressure, and the desired responses are the thermal efficiency and specific energy consumption of the brakes, as well as the concentrations of carbon monoxide, unburned hydrocarbon, and oxides of nitrogen. Root-mean-square error and the coefficient of determination were used to assess the predictive power of the model. Comparatively to Artificial Intelligence and the Response Surface Methodology-Genetic Algorithm model, the results provided by NSGA-II are superior. This is because it achieved a pareto optimum front of 24.45 kW, 2.76, 159.54 ppm, 4.68 ppm, and 0.020243% for Brake Thermal Efficiency, Brake Specific Energy Consumption, Oxides of nitrogen, Unburnt Hydro Carbon, and Carbon monoxide. Combining the precision of ANFIS's prediction with the efficiency of NSGA-optimization II's gives a reliable and thorough evaluation of the engine's settings. The qualitative assessment considered practical aspects and engineering constraints, ensuring the feasibility of applying the parameters in real-world engine applications.

## Introduction

Conventional sources of energy, such as crude oil and coal, have been dwindling at an alarming rate in recent years. This trend is most likely attributable to the increase in population that has been seen around the world^1,2^. Because of this, the transportation industry is now operating in an atmosphere marked by unpredictability. Countries with a higher population are exploring for alternative fuels that have a cheap beginning cost and a comprehensive energy potential^3^. Researchers were encouraged to investigate a feasible, sustainable alternative to diesel that may either completely or partly replace them as a result of the crises that were described above. In earleier bioenergy has shown impecable potential replacement to petro-diesel; nevertheless, high starting prices, poor generation rates, deficiency of knowledge, incorrect physiochemical qualities, and high creation charges taken as a proven to be important challenges for biofuels to become generally embraced^4^. To this point, non-edible oils and waste resources have been identified as a candidate for the role of replacement for crude oil^5^. The production of oil that is not edible needs a huge amount of land, which is not feasible in countries with a high population density per square kilometre^6,7^. If the garbage that has been gathered for conversion in landfills is allowed to react with the rain water, a black liquid known as leachate oil is produced. This leachate oil, if left untreated, will seep under the surface and react with the ground water^8,9^. The majority of the leachate is made up of dissolved and suspended materials, both of which are amenable to being filtered out with the use of appropriate chemical processes^10–12^. Because of the complexities involved in the burning process, treating these oils is not a realistic option. On the other hand, if we take into account the fact that crude oil supplies are expected to run out in the not-too-distant future, it appears like there could be a chance that these oils might be transformed into an alternative motor fuel that is both cost-efficient and environmentally friendly. Although using biodiesel-diesel blends does provide appropriate and acceptable pollution levels, comparison of its performance features with clean diesel fuel indicates levels of performance that are below par. In earlier research, waste oils were combined with nanoparticles in order to improve the biodiesel qualities that were being evaluated. The findings of these evaluations suggested that the oil would have a decreased density and viscosity, in addition to a significant increase in its calorific value. This adds to the advantages it already has to become a possible alternative fuel in the future^13,14^. If these production and mixing nanoparticles parameters are carefully designed through proper modelling under optimum operating conditions, outcome of this will be a reduction in both the cost and time required for biofuel production, which in turn will facilitate its commercialization on a global scale and in industrial contexts^15^.

Recently, biodiesel-based fuels are blended with a variety of nanoparticles presumably to supress the disadvantages associated with using it in diesel engine. Initially distilled water and nanoparticle are amalgamated thoroughly to produce whitish fluid which is later on added in biodiesel. The prime reason of amalgamating nanoparticles is the formation of structured layers in the fuel where these particles might occupy different locations and form heat generating pockets. This facilitates faster combustion due to increase in thermal conductivity of oil. This further reduces the overall delay time providing a wholesome combustion with smoother operation. Also, applying nanoparticles in fuels furnish higher atomization, thereby providing proper mixing as molecules are broken down into little ones with particles embedded between layers.

The use of artificially intelligent (AI) models, which forecast the performance-emission features of petro-diesel engine, is done so in order to get an understanding of the link between the many input parameters of a diesel engine and its many outputs. This effectively cuts down on the number of experimental runs, which in turn cuts down on both operating costs and the amount of time required for the assessment of performance (BTE and BSEC) and exhaust parameters (CO, UBHC and NOx). Through the use of AI in conjunction with various optimization strategies, the predictive models provide, in addition, the optimal combination of inputs that might possibly be generated. Various researchers have used the ANN approach in the course of earlier engine-related investigations in order to cut down on the number of operations and achieve noteworthy results^16^. In addition, a technique known as response surface methodology (RSM) is used in order to provide precise experimental data about the performance and emission characteristics of a petro-diesel engine^17^.

Gopal et al.^18^ evaluated the brake thermal efficiency of alcohol mixed diesel fuels, in order to prepare comparative results with conventional experimental model and ANFIS model. Singh et al.^19^ compared the performance and emission parameters predicted by employing soft computing techniques from blends of Kusum oil. Aghbashlo et al.^20^ employed the ANFIS model in order to forecast output values and develop an objective function on the basis of the process parameters. Odibi et al.^21^ employed the ANFIS model to estimate the exergy efficiency for developing biodiesel from waste cooking oil. Hosoz et al.^22^ estimated the performance parameters of a diesel engine by employing the ANFIS model on a single cylinder engine with input conditions as speed and load. The literature survey highlights the prevalent use of artificially intelligent (AI) models, particularly ANN and ANFIS, in forecasting performance-emission features of petro-diesel engines, leading to reduced experimental runs, operating costs, and assessment time. However, the research gap lies in the limited application of AI models to combine alternative fuels with nanoparticles, the lack of comprehensive comparative studies on optimization strategies, inadequate consideration of engine design parameters with the use of nanoparticles in fuel, limited generalization across different engine types and neglect of uncertainty analysis,. Addressing these gaps will enhance the applicability and reliability of AI models in optimizing diesel engine performance, emissions, and design. Henceforth the above literature has clearly established the ANFIS method as an accurate predicting model which integrates the advantages of feed forward calculation for an outcome, back propagation learning capacity of ANNs and human-like reasoning style of fuzzy logic.

In light of the aforementioned developments, the scholars have come up with the following viewpoints, which are outlined below:

- Biodiesel derived from landfills, when combined with nanoparticles and applied to diesel engines, has the potential to successfully improve performance parameters while simultaneously lowering emissions from diesel engines. This is because the application of biodiesel derived from landfills boosts up the performance parameters of a diesel engine.
- The use of waste oils as a potential solution is shown to be a workable and practicable alternative on account of their simple accessibility and their little impact on the surrounding natural environment.
- The investigation of engine outputs for a variety of nanoparticles and their concentrations while using soft computing approaches such as ANFIS, ANFIS-GA, and ANFIS-NSGA-II has never been discussed in any of the prior literature.
- Previous research, conducted in other subfields of thermal engineering, has shed light on the significance of integrating exceptional forecast representations with optimization systems to produce exact petro-diesel engine variables while simultaneously reducing the amount of effort, cost, labour, time, and energy required.

To the authors fullest knowledge very few research work was available online regarding AI based optimization of diesel engine. Furthermore, application of meta-heuristic techniques in diesel engine still remains an unexplored domain while generating outputs for biodiesels. The authors keeping in mind the above trend were motivated to perform a performance and exhaust analysis of biodiesel while varying for prime input parameters with the aid of multiple hybrid metaheuristic techniques.

The current research applied the ANFIS model as output prediction and further integrated the objective functions by optimizing the outcomes with the aid of genetic algorithms (GA) and its higher version (NSGA-II). As a result, the influence of a number of input factors, such as load, blend%, nanoparticle concentration (NPC), and injection pressure (IP), may be investigated using these models in a way that is efficient and inexpensive. When the expected answers of ANFIS were put up against the results gained through experimental assessments, it was discovered that ANFIS's predictions were fairly accurate. The ANFIS-GA model developed is remarkably capable of replicating the conventional method for engine output evaluations with low error rate. Furthermore, another version of genetic algorithm called non-sorting genetic algorithm (NSGA-II) is employed to test its viability in diesel engine, thereby comparing output responses for all models.

This research is groundbreaking in its use of prognostic systems (ANFIS) and optimization practices (GA and NSGA-II) to enhance the performance of engines and decrease emissions by utilizing nanoparticles in landfill oils. Notably, no prior studies have investigated nanoparticle variation by prediction models in this area. The implementation of a novel model in the biofuel industry minimizes the number of engine runs required to achieve accurate predictions through rigorous data training, testing, and validation, underscoring the innovative and precise nature of this study. In the realm of diesel engines, the introduction of soft computing methods in conjunction with optimization approaches will provide results that are revolutionary.

The objectives of this research are as follows: Firstly, to conduct a performance and exhaust analysis of biodiesel by varying prime input parameters, namely load, blend %, nanoparticle concentration (NPC), and injection pressure (IP), using hybrid metaheuristic techniques. Next, to utilize the ANFIS model for output prediction and integrate objective functions for optimizing outcomes using Genetic Algorithms (GA) and its higher version (NSGA-II). Furthermore, to investigate the efficiency of the ANFIS-GA model in evaluating engine outputs by comparing its predictions against experimental assessments. Finally, to assess the viability of the non-sorting Genetic Algorithm (NSGA-II) in the context of diesel engine performance and exhaust analysis, while comparing its output responses with other models employed in the study. By achieving these objectives, the research aims to contribute to the advancement of AI-based optimization techniques for diesel engines and the understanding of biodiesel performance characteristics.

## Materials and methods

In order to get the best possible outcome, it is necessary to carry out preliminary functional operations such as specifying the input and output variables in advance. The experimental data set is created by varying four parameters: load (LD), blend percentage (BP), nanoparticle concentration (NPC), and injection pressure (IP). The proposed input will be measured against this standard to ensure it achieves the highest possible thermal efficiency in the brakes, the lowest possible specific fuel consumption, and the cleanest possible exhaust gas emissions (including CO, NOx, and UBHC). In this study, we provide several hybrid methods for comparing the experimental and anticipated data, which we do in four stages. The steps involved are as follows: (i) aggregating the acquired experimental data and grouping it based on training and testing; (ii) selecting the best performance model in the ANFIS & RSM data structure for assessing the performance and exhaust emission parameters; (iii) integrating the results of the ANFIS & RSM model with the GA and NSAGA-II optimisation technique; and (iv) finally, generalising the optimised results, including maximum BTE, minimum BSEC (CO, NOx and UHBC).

### Data compilation

The diesel engine test rig that was fueled with landfill food waste oils served as the source of the experimental dataset that was used in the development of the prediction ANFIS model. These oils were linked together with two nanoparticles at the same time, namely aluminium oxide (Al_2_O_3_) and copper oxide (CuO). The RSM random modelling approach was used to construct the datasets for the purpose of producing outcome responses. This technique resulted in the generation of 60 distinct datasets for foolproof data interpretation. On a diesel engine, performance and emission characteristics were analysed for both mixes, and table of data was generated based on various nano-additive absorptions for varying blend percentages, engine loads, and injection pressures. The following parts include a comprehensive explanation of the procedures that were carried out during the creation of the data, the assessment, and the analysis. The specifications for measuring the outcomes of the system is provided in Table 1.

**Table 1:** Specifications of measuring instruments.

Parameter	Apparatus	Measuring range	Equipment model
Brake power	Dynamometer	0–1000 kW	DynoTech 2000
Torque	Dynamometer	0–5000 Nm	TorqMaster Pro 5000
Fuel consumption	Fuel flow meter	0–1000 L/h	FlowTech FLM-1000
Exhaust gas temp	Thermocouple	0–1200 °C	TempProbe TC-1200
NOx emissions	NOx analyzer	0–1000 ppm	AVL gas analyser
CO emissions	CO analyzer	0–10%	AVL gas analyser
UBHC emissions	Hydrocarbon analyzer	0–1000 ppm	AVL gas analyser
Cylinder pressure	Pressure transducer	0–200 bar	PressSureMaster PT-200
Injection pressure	Pressure transducer	0–2000 bar	InjexiTech IPX-2000

### Materials preparation and biodiesel production analysis

**Figure 1:**
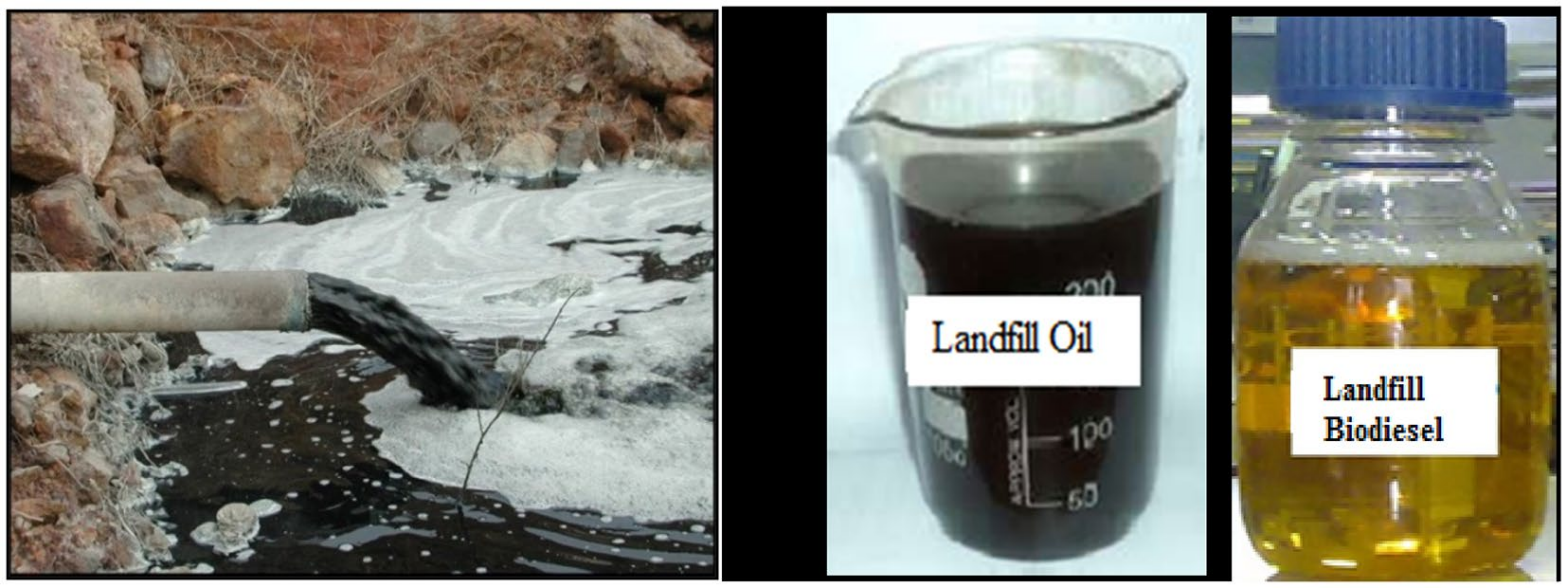
Generation of landfill oils at a landfill site, landfill oil and landfill biodiesel sample.

This section in particular covers the process of acquiring leachate oil and nanoparticles (Al_2_O_3_ and CuO) and further details the procedure that is involved in the manufacture of different mixes. The primary source of the raw leachate oil used in this study was the local landfill facility at Timarpur, which can be found in New Delhi and is represented in Fig. 1. The collected leachate oil was carried into the laboratory, where it was heated to a temperature of 120 degrees Celsius and filtered in order to remove any traces of moisture that may have been present. We purchased excess chemicals from the neighbourhood market, including methanol with a purity of 99%, potassium hydroxide with a concentration of 96%, and an indicator containing phenolphthalein. Powdered aluminium oxide and copper oxide were purchased from a local trader at the chemical market in Ghaziabad, Uttar Pradesh. The Free fatty acid (FFA) level was assessed to be much greater than 2%, which meant that the raw oil needed to go through a transesterification process that consisted of two stages in order to be converted into biodiesel. The experiment was carried out in a glass beaker, and it was carried out in such a manner that a solution of KOH and methanol were fully mixed with the assistance of an ultrasonic machine that comprises of an ultrasonic horn for effective mixing. In the combination containing methoxide, further amounts of landfill oil and hydrobromic, which is an acid catalyst, were combined, and the frequency of mixing was increased. The end result was a mixture of glycerine and biodiesel, which could be seen as two distinct layers after it had been processed. While the quantities of acid and oil were held at a constant ratio of HBr/oil = 20/200 (w/w), the ratio of methanol to water was kept at a constant level of 200 grammes per 400 millilitres^23^. When nanoparticles are added to fuels, the end result is often an improvement in the qualities of the fuel, such as an increase in the amount of excess oxygen and an expansion of the contact surface area. Because nanoparticles are often insoluble in biofuels, successful amalgamation frequently necessitates the inclusion of an extra chemical reaction as well as a procedure that amplifies the amount of energy available. The transformation of particles made of metal into nanofluids, which are readily miscible with biodiesels, is the primary transformation that takes place. The process begins with the treatment of metal particles with distilled water, and it is followed by the transfer of the mixture into an ultrasonic reactor for a predetermined period of time. During the course of this investigation, aluminium oxide and copper oxide are weighed before being mixed with water that has been distilled, and then the mixture is subjected to an ultrasonic reactor operating at 90–100 kHz for twenty minutes^24^. Nanoparticles of Al_2_O_3_ and CuO that were employed in the study have a size of thirty nanometers (nm). Nanoparticles with proper surface modifications and well-chosen surfactants can enhance stability by preventing agglomeration and sedimentation. Secondly, maintaining a controlled and uniform dispersion of nanoparticles in the fuel matrix is essential. This can be achieved through advanced mixing techniques and optimization of processing parameters. Thirdly, understanding the impact of nanofuel properties on engine performance is vital. Thorough testing and evaluation of the nanofuel's combustion characteristics, emissions, and engine durability are necessary to ensure compatibility and stability during engine operation. After that, the milky nanofluid is mixed with biodiesel using a magnetic stirrer at a speed of 1800 revolutions per minute while Span 80, a surfactant, is also present in the mixture. The final composition of the test mix for the aluminium biodiesel (ABD) and the copper biodiesel (CBD) was 92% biodiesel, 5% nanofluid, and 3% surfactant on a volume basis^25^.

### Setup of test engine

**Figure 2:**
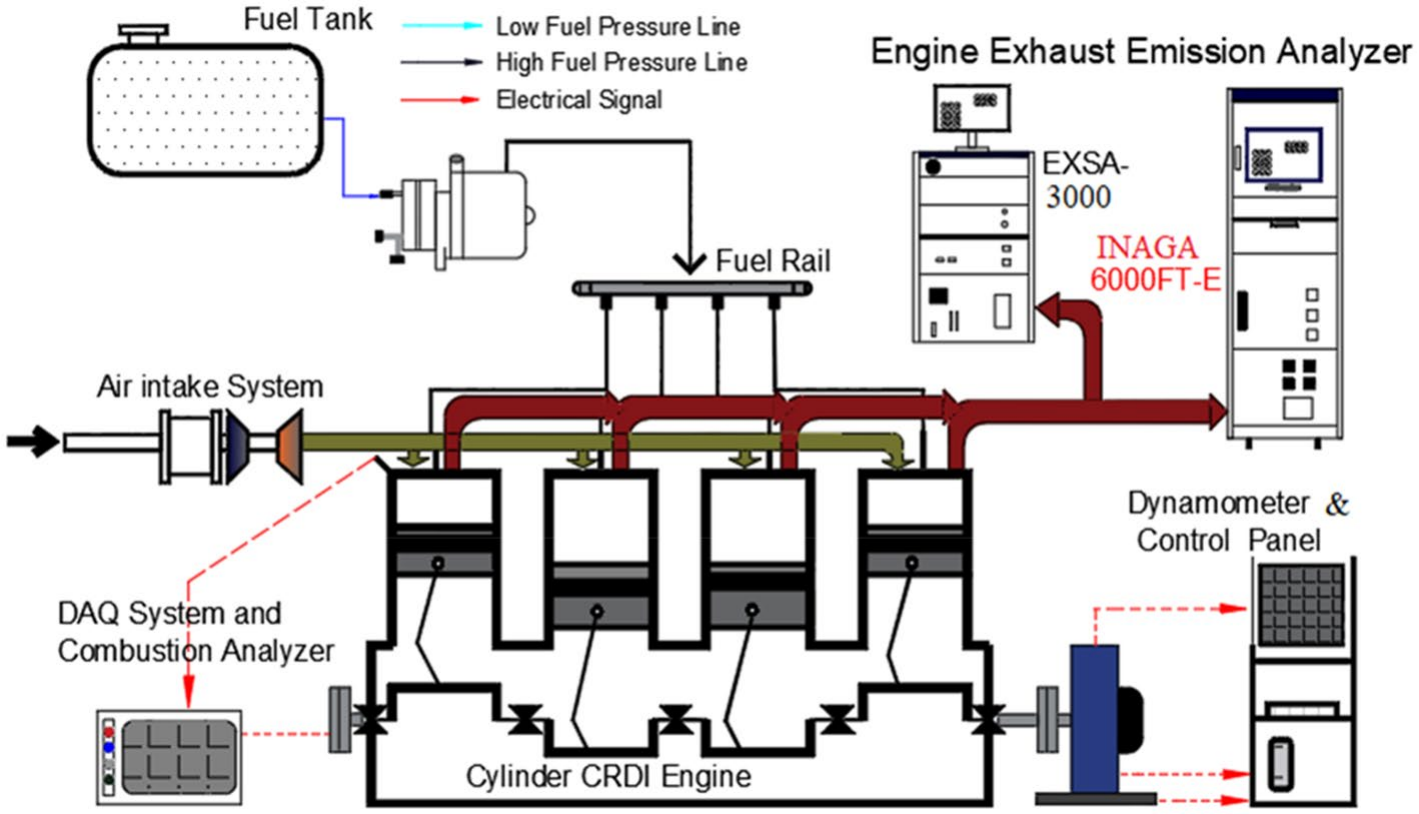
Experimental setup of PETTER-AV engine.

In Fig. 2, the engine configuration that was utilised to calculate the performance and emission characteristics for biodiesel-based fuels is provided for the reader's convenience. The CRDI diesel engine served as the testing ground for the first experiments. The eddy current dynamometer is used to measure and adjust the amount of load that is placed on the engine. The voltmeter and the current metre are both components of the resistive type load panel. Controlling the engine torque in the range of 0 to 18 kg required the employment of a diesel engine and an eddy-current dynamometer in conjunction with each other. A thermocouple of the K type was used in order to get an accurate reading of the exhaust gas's temperature. The exhausts from the engine predominantly consist of UBHC, CO, and NOx emissions, all of which were shown on the screens of the gas analysers labelled "AVL Di Gas 444". The many technical particulars of the engine configuration are outlined in Table 2, which can be found below.

**Table 2:** Mechanical particulars of the petro-diesel engine.

S. no.	Module	Description
1	Main engine model	KIRLOSKAR
2	Engine design	4-stroke, petro-diesel engine
3	Bore of engine cylinder	90 mm
4	Length of cylinder	130 mm
5	Power production	17 BHP at 4000 rpm
6	Compress ability	18:1

### Physio-chemical analysis of produced test blends

In order to further examine the practicability of using the produced fuel in a CI engine, varied combinations of petro-diesel and nano-particles were added to the mixture. As can be shown in Table 3, the physio-chemical parameters of leachate oil are quite similar to those of regular diesel, which suggests that it would be suitable for use in an engine. The advantage of the fuel in the injection process was further increased by other advantages, such as a reduction in viscosity. A capillary viscometer was used in order to determine the thickness of the fuel mixtures. A bomb calorimeter equipment was used to measure the calorific value of the fuel while a hydrometer was used to estimate the density of the fuel. As can be seen in Table 3, the final test blends of aluminium oxide biodiesel (abbreviated as ABD) and copper oxide biodiesel (abbreviated as CBD) were determined to be in compliance with the ASTM criteria, as shown by the physiochemical parameters of the blend.

**Table 3:** Comparison for physiochemical properties of the test fuels.

Properties	ABD	CBD	LFB test fuel	Diesel	ASTM limit
Density at 15 °C (kg/m^3^)	888	893	975	841	860–900
Kinematic viscosity (cSt)	3.64	3.52	3.99	4.56	2.52–7.5
Calorific value (MJ/kg)	47.5	45.5	39.12	44.85	Min. 33
Flash point (°C)	69.83	65.71	70.45	51	Min. 130
FFA (%)	–	–	0.77	0.0014	Max. 2
Fire point (°C)	59	57	47	58	Min. 53

### Application of response surface methodology technique

An early attempt to tackling the problem of connecting the input parameters with the output parameters is offered by the RSM method. In order to calculate the outputs and decide which equation best matches them, a specialised CCRD design was used, and sixty test runs were performed. In addition to this, the built architecture provides newly calculated extreme values (both low and high) for each variable^26–28^. Data gathered from previous research is used to create the input variables, and the outcomes of experiments are used to validate whether or not such variables are feasible. The ranges that were created for the input parameters had a significant impact on the output responses up to a certain point, beyond which the effects were much less significant^29^. The amount of load that was provided to the engine was adjusted between 20 and 100%, and the fuel was blended up to 20%. The engine was subjected to a maximum injection pressure of 220 bars, and the concentration of nanoparticles was varied between 0 and 20%. When the load was less than 20%, the results were trivial and not helpful to the study at all^30^. The calorific value of the gasoline decreased when it was blended at a higher percentage than 20%, which rendered the running of the engine impossible. The concentration of nanoparticles was capped at 20% due to the fact that any higher would have caused the fuel to become very viscous. Injection pressures higher than 220 bar needed a modification to the design of the engine in order to achieve efficient functioning, which is not an option that can be pursued. The experiment was carried out using two distinct nanoparticles, namely Al_2_O_3_ and CuO, in order to determine which of the two is superior in terms of the output responses. Two measurements were obtained at the same time for a certain set of input variables in order to get values that were foolproof and included the least amount of uncertainty possible. In the end, their average value was the answer that was taken into consideration for the next stage of the research. After deriving the fits equation for each output function, it was then input into the optimization models (GA and NSGA) as described in Table 4. This was done while the system was operating using petro-diesel as its fuel.

**Table 4:** Level of experimental parameters.

S. no.	Input parameters	Unit	Level 1	Level 2	Level 3	Level 4	Level 5
1	Load (L) (%)	%	20	40	60	80	100
2	Blend (B) (%)	%	0	5	10	15	20
3	Injection pressure (IP) (bar)	Bar	180	190	200	210	220
4	Nano particles concentration (NPC) (%)	%	0	5	10	15	20

The analysis makes use of a number of different control variables, numerical and coded data, and a CCRD array that was built specifically for it. In all, there were sixty different runs. A summary of the findings obtained from sixty separate test runs conducted using a variety of engine input parameters.

The analysis contains four variables, each of which is shown in Table 4 along with their respective ranges. The numerical values that were used in the array that was specifically developed for your application and consisted of a total of 60 runs Table 5 presents a summary of the findings obtained from sixty separate test runs conducted.

**Table 5:** Experimental outcomes from diesel engine.

Input conditions					ABD					CBD			
Trial	Appl	Mix %	IP	NA	BTE	BSEC	UBHC	CO	NOx	BTE	BSEC	CO	NOx
1	20	15	180	5	16.17	3.7149	7.9099	0.0143	170.3	15.561	4.32	0.018	197
2	40	5	190	0	13.93	3.5014	8.7676	0.0123	172.5	13.406	4.1	0.016	200
3	60	10	200	20	20.89	2.7328	6.0992	0.0185	145.3	20.11	3.2	0.024	168
4	80	0	210	15	18.65	2.5193	7.4334	0.0165	147.6	17.955	2.95	0.021	171
5	100	20	220	10	22.38	3.3306	5.9086	0.0198	204.3	21.546	3.9	0.025	237
6	20	5	180	5	14.18	3.3733	8.6723	0.0125	152.1	13.646	3.95	0.016	176
7	40	15	190	10	18.4	3.416	7.0522	0.0163	168	17.716	4	0.021	195
8	60	10	200	0	15.92	3.5868	8.0052	0.0141	190.7	15.322	4.2	0.018	221
9	80	0	210	15	18.65	2.5193	7.7193	0.0165	147.6	17.955	2.95	0.021	171
10	100	20	220	20	24.87	2.9036	4.9556	0.022	181.6	23.94	3.4	0.028	210
11	20	0	180	0	11.94	3.416	9.53	0.0106	154.4	11.491	4	0.013	179
12	40	5	190	5	15.17	3.2879	8.2911	0.0134	161.2	14.603	3.85	0.017	187
13	60	10	200	10	18.4	3.1598	7.0522	0.0163	168	17.716	3.7	0.021	195
14	80	15	210	20	22.88	2.8182	5.8133	0.0202	163.4	22.025	3.3	0.026	189
15	100	20	220	15	23.63	3.1171	8.1005	0.0209	193	22.743	3.65	0.027	224
16	20	0	180	0	11.94	3.416	9.53	0.0106	154.4	11.491	4	0.013	179
17	40	10	190	5	16.17	3.4587	7.9099	0.0143	170.3	15.561	4.05	0.018	197
18	60	5	210	10	17.66	3.0317	7.5287	0.0156	161.2	16.997	3.55	0.02	187
19	80	15	220	15	21.89	3.0744	6.3851	0.0194	177.1	21.067	3.6	0.025	205
20	100	20	200	20	24.37	2.8182	4.765	0.0216	177.1	23.461	3.3	0.027	205
21	20	0	190	0	12.19	3.4587	9.4347	0.0108	156.6	11.731	4.05	0.014	181
22	40	5	200	5	15.42	3.3306	8.1958	0.0136	163.4	14.843	3.9	0.017	189
23	60	10	180	10	17.91	3.0744	7.2428	0.0158	163.4	17.237	3.6	0.02	189
24	80	15	210	15	21.64	3.0317	6.2898	0.0191	174.8	20.828	3.55	0.024	203
25	100	20	220	20	24.87	2.9036	4.9556	0.022	181.6	23.94	3.4	0.028	210
26	20	0	180	0	11.94	3.416	9.1488	0.0106	154.4	11.491	4	0.013	179
27	40	15	200	5	17.41	3.6722	7.4334	0.0154	181.6	16.758	4.3	0.02	210
28	60	10	190	10	18.16	3.1171	7.1475	0.0161	165.7	17.476	3.65	0.02	192
29	80	20	210	15	22.63	3.2025	5.9086	0.02	183.9	21.785	3.75	0.025	213
30	20	5	220	20	18.9	2.9036	7.2428	0.0167	127.1	18.194	3.4	0.021	147
31	100	0	190	0	15.17	2.9463	8.2911	0.0134	183.9	14.603	3.45	0.017	213
32	40	5	180	5	14.92	3.2452	7.624	0.0132	158.9	14.364	3.8	0.017	184
33	60	10	200	10	18.4	3.1598	7.0522	0.0163	168	17.716	3.7	0.021	195
34	80	15	210	15	21.64	3.0317	6.2898	0.0191	174.8	20.828	3.55	0.024	203
35	100	20	220	20	24.87	2.9036	6.0992	0.022	181.6	23.94	3.4	0.028	210
36	20	0	180	20	16.91	2.562	7.624	0.015	109	16.279	3	0.019	126
37	40	5	190	5	15.17	3.2879	8.2911	0.0134	161.2	14.603	3.85	0.017	187
38	100	10	220	5	21.64	3.2025	7.1475	0.0191	197.5	20.828	3.75	0.024	229
39	80	15	210	10	20.39	3.2452	6.7663	0.018	186.1	19.631	3.8	0.023	216
40	60	20	200	0	17.91	3.9284	7.2428	0.0158	208.8	17.237	4.6	0.02	242
41	20	0	200	0	12.44	3.5014	9.3394	0.011	158.9	11.97	4.1	0.014	184
42	40	5	190	5	15.17	3.2879	8.2911	0.0134	161.2	14.603	3.85	0.017	187
43	100	10	180	10	19.4	2.8182	6.671	0.0172	177.1	18.673	3.3	0.022	205
44	60	15	210	15	20.89	3.1598	6.2898	0.0185	168	20.11	3.7	0.024	195
45	80	20	220	20	24.12	3.0317	7.4334	0.0213	174.8	23.222	3.55	0.027	203
46	20	0	180	0	11.94	3.416	9.53	0.0106	154.4	11.491	4	0.013	179
47	40	5	200	5	15.42	3.3306	7.2428	0.0136	163.4	14.843	3.9	0.017	189
48	60	10	190	15	19.4	2.9036	6.671	0.0172	154.4	18.673	3.4	0.022	179
49	80	15	210	10	20.39	3.2452	6.7663	0.018	186.1	19.631	3.8	0.023	216
50	100	20	220	20	24.87	2.9036	4.9556	0.022	181.6	23.94	3.4	0.028	210
51	20	0	190	0	12.19	3.4587	9.4347	0.0108	156.6	11.731	4.05	0.014	181
52	40	5	180	5	14.92	3.2452	8.3864	0.0132	158.9	14.364	3.8	0.017	184
53	80	10	200	10	19.15	3.0317	7.0522	0.0169	174.8	18.434	3.55	0.022	203
54	60	15	220	15	21.14	3.2025	6.3851	0.0187	170.3	20.349	3.75	0.024	197
55	100	20	210	20	24.62	2.8609	4.8603	0.0218	179.3	23.701	3.35	0.028	208
56	20	5	180	0	12.93	3.5868	9.1488	0.0114	163.4	12.449	4.2	0.015	189
57	40	0	190	5	14.18	3.1171	8.6723	0.0125	152.1	13.646	3.65	0.016	176
58	60	10	200	10	18.4	3.1598	7.0522	0.0163	168	17.716	3.7	0.021	195
59	80	15	210	20	22.88	2.8182	5.8133	0.0202	163.4	22.025	3.3	0.026	189
60	100	20	220	15	23.63	3.1171	5.4321	0.0209	193	22.743	3.65	0.027	224

### Modelling and optimization

**Figure 3:**
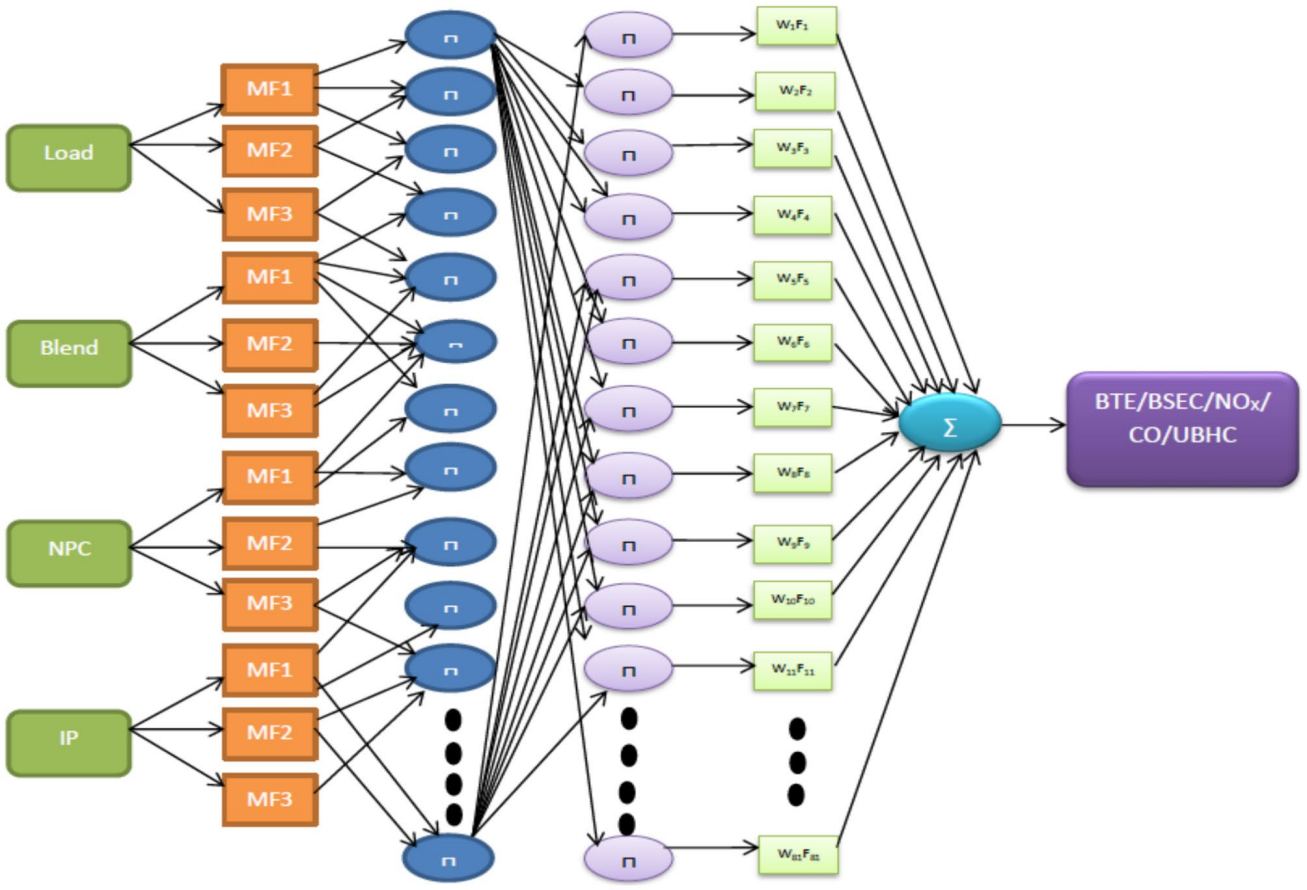
Framework of ANFIS model.

The primary model employed in this study was the Takagi–Sugeno artificial neuro-fuzzy interface system of the first order approach that was used for the research (ANFIS). In order to assess the performance and exhaust characteristics, the experiment was designed on the basis of the model described above and shown in Fig. 3. These results are being taken into consideration as the primary goal functions for this study. Previous research have already created models that are conceptually comparable to this one. However, when applied to thermal engineering applications, these models were complicated, time-consuming, and erroneous owing to the restricted, nonlinear, and uncertain dataset^31,32^. Recently, ANFIS models have acquired a large amount of popularity as a result of their capacity to develop effective fuzzy rules, which enables them to facilitate efficient surface plots between a variety of input and output responses. Practically speaking, there is an urgent demand to adopt such artificially intelligent procedures in engine performance assessments since this methodology may create speedier and more accurate outputs, therefore becoming an ideal alternative way to the current experimental techniques. These approaches are able to provide results that are more reliable and accurate than their predecessors. The general model of ANFIS is made up of six primary layers, starting with the initial layer of input parameters, then moving on to the fuzzification layer, rule consequent layer, rule strength normalisation layer, rule consequent layer, and lastly the rule inference layer^33^. The existence of both the Fuzzy Theory and membership frameworks may be inferred from the successful construction of a workable ANFIS structure. The collection of data was made possible by the creation of five distinct FIS models for objective functions, which were denoted by the acronyms BTE, BSEC, CO, and NOx respectively. A total of approximately sixty sets of input variables and data patterns were generated as a result of the experiments. These were then divided at random into two subsets, namely, forty-five (representing approximately seventy-five percent) and fifteen (representing approximately twenty-five percent) sets of data, which were used for training and testing the ANFIS models, respectively. Table 6 provides an explanation of the model's foundation, which may be found in the single ANFIS.

**Table 6:** ANFIS framework for training the diesel engine-based model.

Over-all quantity of nodes	203
Quantity of linear limitations	104
Amount of non-linear strictures	27
Sum of training information pairs	51
Amount of rules that are fuzzy	99
Relationship role	Triangular

In order to develop a variety of reactions via modelling, the following ANFIS equations were implemented.

#### Layer 1: fuzzification layer

1 $${Q}_{1,i}={\mu }_{{A}_{i}}(x) , for\, i=\mathrm{1,2} \,\, or;$$

2 $${Q}_{1,j}={\mu }_{{B}_{j}}(y) , for\, j=\mathrm{1,2};$$

3 $${\mu }_{{A}_{i}}(x) =\frac{1}{1+{\left[{\left(\frac{ x - {c}_{i}}{{a}_{i}}\right)}^{2}\right]}^{{b}_{i}}}$$

#### Layer 2: product layer

4 $${Q}_{2,i}=\overline{{w}_{i}} = {\mu }_{{A}_{i}}(x) {\mu }_{{B}_{i}}(y), for\, i=\mathrm{1,2};$$

#### Layer 3: normalized layer

5 $${Q}_{3,i}=\overline{{w}_{i}} =\frac{{w}_{i}}{{w}_{1}+ {w}_{2}}, for\, i= \mathrm{1,2}$$

#### Layer 4: defuzzied layer

6 $${Q}_{4,i}= \overline{{w}_{i}} {f}_{i} = \overline{{w }_{i}} ( {p}_{i}x + {q}_{i}y + {r}_{i}), for\, i=\mathrm{1,2};$$

#### Layer 5: total output layer

7 $${Q}_{5,i}= overall\, output = {\sum }_{i}\overline{{w}_{i}} {f}_{i} =\frac{ {\sum }_{i}{w}_{i} {f}_{i}}{{\sum }_{i} {w}_{i}}$$

8 $$f=\frac{{w}_{1}}{{w}_{1}+ {w}_{2}}{f}_{1}+\frac{{w}_{2}}{{w}_{1}+ {w}_{2}}{f}_{2}$$

9 $$f= \overline{{w }_{1}}( {p}_{1}x + {q}_{1}y + {r}_{1}) +\overline{{w }_{2}} ({p}_{2}x + {q}_{2}y + {r}_{2})$$

10 $$f=({\overline{{w }_{1}}p}_{1}x + \overline{{w }_{1}}{q}_{1}y + \overline{{w }_{1}}{r}_{1}) +(\overline{{w }_{2}}{p}_{2}x + \overline{{w }_{2}}{q}_{2}y + \overline{{w }_{2}}{r}_{2})$$

In most cases, the process of modelling and optimising an engine based on its features starts with the creation of a precise fitness function that is appropriate for the level of difficulty of the problem statement. The traditional approaches that are used to construct objective function for a number of input and output parameters need a significant amount of time and effort to complete. However, since it is able to produce the data without the need for any prior model history, the ANFIS methodology is able to provide an adequate objective function. This is the primary reason why this method is recommended. Utilizing a genetic algorithm in the processing of output replies allows for further refining of estimations and predictions that have been derived using the ANFIS method, which results in improvements in both accuracy and efficiency.

**Figure 4:**
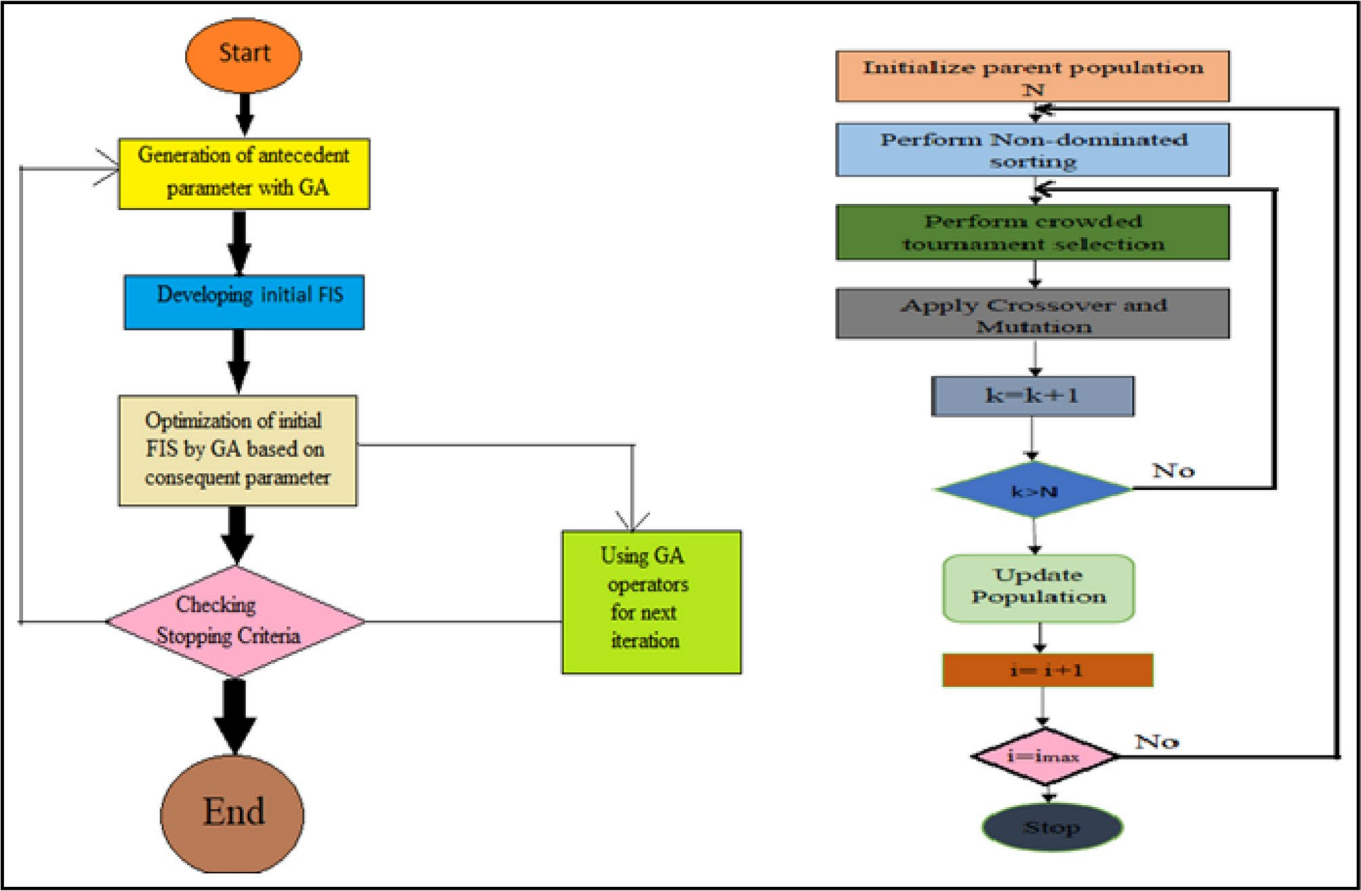
Algorithm of RSM-GA (left) and ANFIS-NSGA-II algorithm (right).

When it comes to multi-objective optimization, it is often seen that the outputs of the ANFIS approach are trapped inside the local optima, which indicates that the technique may not be 100% correct^34^. In addition to this, the model construction is made more difficult by the contradictory results. Hybrid approaches, such as the genetic algorithm and its many extensions, are used in order to optimise the problem in a way that is both prompt and effective. This is done in order to circumvent the challenges presented by the issue's The implementation of a GA algorithm that randomly searches for solutions makes for replies that are both economic and competent. The GA is a kind of adapt-capable combinatory exploration procedure that operates based on the fundamental concept of biotic development. This means that the variations are generated depending on the mixture of the parent and kid. The dataset is used by the model to construct the optimal combination of inputs that will result in the best results, and the framework for these outputs can be found in Tables 6 and 7. Previous studies have shown that GA is more effective than other multivariate techniques, which take a longer amount of time to produce results even when using comprehensive nonparametric strategies^35,36^. These studies compared GA to other multivariate techniques and found that GA produced more accurate results in a shorter amount of time. In light of this, making use of a GA weighted sorting approach in RSM produced predictions results in a reduction in the amount of uncertainty. The RSM-GA algorithm's flowchart may be seen in Fig. 4, which is given below for convenience.

**Table 7:** GA algorithm framework.

Category of assortment technique	Roulette wheel
Populace gauge	85
Repetitions	3400
Switch-over (%)	0.85
Alteration proportion (%)	0.85
Equal of alteration	0.9
Assortment weight	12

To enhance its performance in meeting the general norms for petro-diesel machines, the model undergoes further optimization using the NSGA-II algorithm. This algorithm, known for its elitist structure, has demonstrated success in engineering applications for multi-function optimization, top to quicker meeting and priority-based categorization^37,38^.

In the optimization process, the input variables are evaluated for Pareto optimality and then fed into the Fuzzy Interface System (FIS) framework^39^. Within the FIS, membership functions are defined for each input value. The NSGA-II algorithm is then applied to this framework to achieve multi-objective optimization for all outcomes listed in Table 8.

**Table 8:** NSGA-II optimization process requirements and parameters.

Parameter	Value
Objectives	Max BTE, Min BSEC, Min NOx, Min CO and Min UBHC
Variables	X_1_ = load, X_2_ = blend percentage, X_3_ = ignition pressure, X_4_ = nanoparticle concentration
Bounds	20 ≤ X_1_ ≤ 100, 0 ≤ X_2_ ≤ 20, 180 ≤ X_3_ ≤ 220, 0 ≤ X_4_ ≤ 20
Populace type	Double vector
Populace size	300
Collection function	Tournament
Cusp portion	0.8
Transformation portion	0.1
Ending norms	Generations: 1000/stall generations: 100

**Figure 5:**
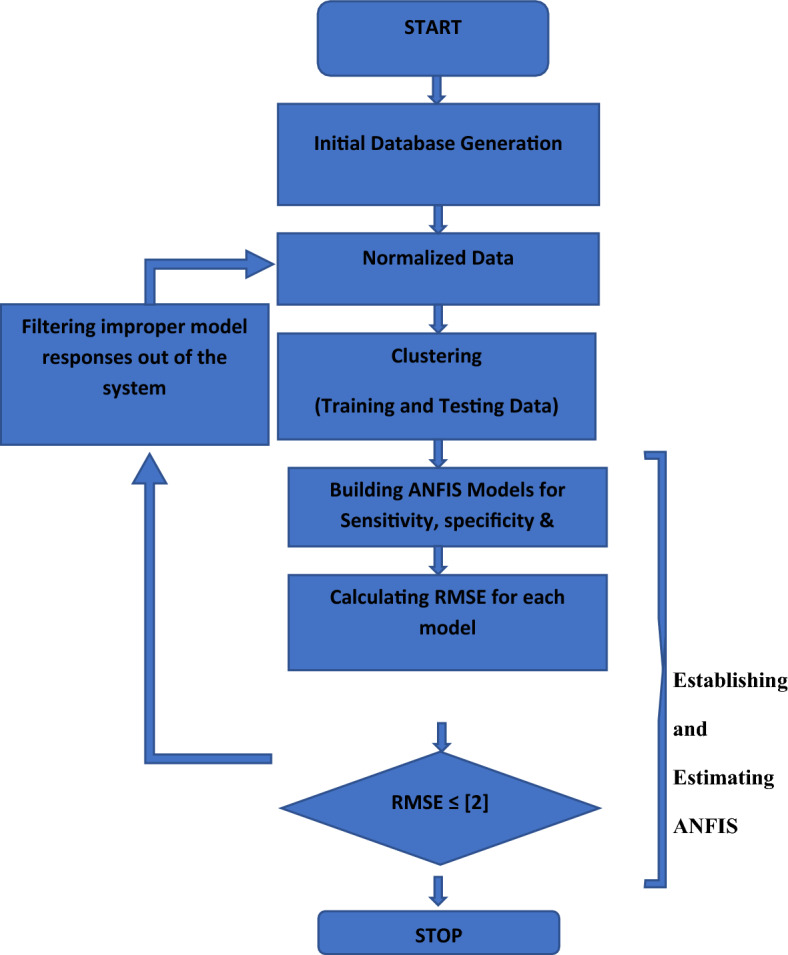
Flowchart for the applied prediction model of ANFIS.

Primary aim of developing an optimization model is to maximize the BTE and minimize BSEC, NOx, UBHC and CO simultaneously A detailed flowchart is presented below Fig. 5 highlighting the steps involved in ANFIS-NSGA-II approach in generating a multi-objective output for performance and exhaust emission of landfill waste biodiesel.

**Figure 6:**
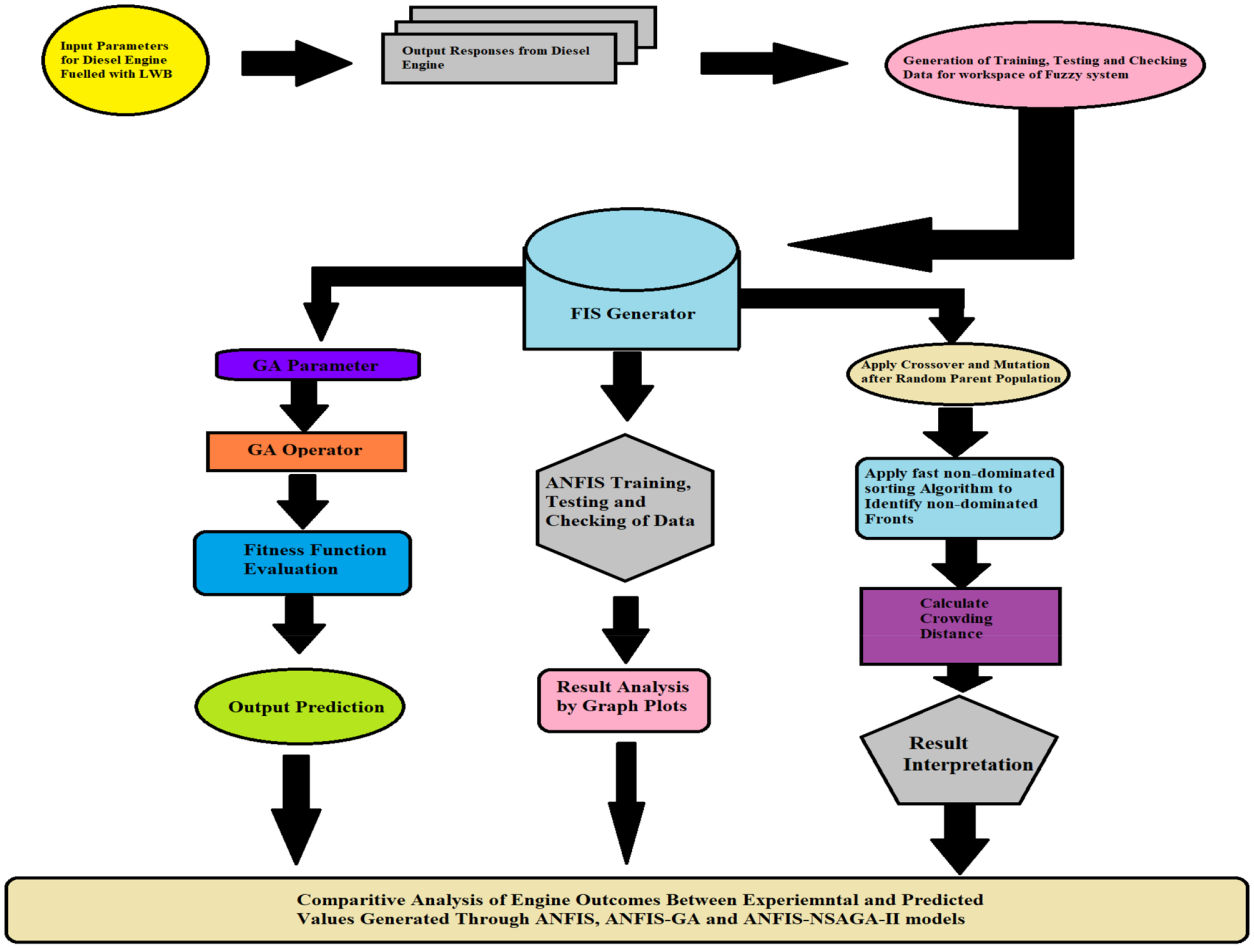
Combined flow chart for various processes accompanied in this research.

A combined flow chart representing the various processes accompanied in this research is shown in Fig. 6.

The NSGA-II algorithm was utilized to optimize the interdependency matrix and generate optimal responses. The following steps were undertaken in the application of NSGA-II algorithm:

- Step one: Random primary population is created of size N.
- Step two: The population formed in step one sorted by means of fast non-dominated sorting until the entire population is classified into numerous fronts.
- Step three: Crowding distance task is performed for every evaluation and crowded tournament assortment is allocated. This picks a combination at a improved rank if the combinations belong to various fronts or a answer with a higher crowding distance if they belong to the identical front.
- Step four: Crossover and mutation is applied to the parent population obtained above to yield child population. To generate new offspring’s, simulated binary crossover (SBX) operator and polynomial mutation operators are used.
- Step five: The parent and child population are joint together to yield a population of size 2N.
- Step six: Stopping criteria is checked. If the Pareto optimal front is accomplished, then algorithms is stopped else repeat and move to step two.

All major data applied and generated in the ANFIS models. Statistical tools such as the coefficient of determination (R^2^) could be used to elucidate the discrepancies in the developed model and mean-squared error (RMSE) provided in Eqs. (11) and (12) respectively.

11 $$RMSE=\sqrt{\frac{1}{N}\sum_{i=1}^{N}{\left({P}_{i}-{E}_{i}\right)}^{2}}$$

12 $${R}^{2}=1-\frac{\sum_{i=1}^{N}\left({P}_{i}-{E}_{i}\right)}{\sum_{i=1}^{N}\left({P}_{i}-{E}_{m}\right)}$$

where, $${E}_{m}=\frac{\sum_{i=1}^{N}{P}_{i}}{N}$$

RMSE = Root Mean Square Error, R^2^ = Fraction of Variance, P_i_ = Forecast value obtained from modelling, E_i_ = Experimental value generated, E_m_ = Mean of the predicted values generated from models, N = Available Data, i = Trial run value need to be calculated.

### Output responses and their measurement

In this research, output responses were measured by employing measuring apparatuses, chemical formulas and empirical relations depending upon the type of response. In the present study six output parameters were considered and their measurement method is described as follows:

#### Brake thermal efficiency (BTE)

This maybe defined as the ratio of the applied brake power (BP) attained at the crankshaft to total energy (E) available to diesel engine for combustion process as shown in Eq. (13).

13 $$\eta = \frac{BP}{E}$$

where, *η* is the brake thermal efficiency (in %); *BP* is the Brake power (in kW) and *E* is the fuel energy (in kW).

#### Brake specific energy consumption (BSEC)

This quantity is a hypothetical tool which indicates the total energy required from the fuel to produce unit power. Over the years it has become a powerful tool and somewhat replaced the BSFC value. Fuels are compared for fuel efficiency with this tool only. BSEC is the calorific value (CV) times brake specific fuel consumption (BSFC) and used to prepare a comparison among various fuels. The specific energy consumption is a more accurate estimate in comparison to specific fuel consumption. It is given by Eq. (14)

14 $$BSEC \, = \, CV \, \times \, BSFC$$

where, *CV* is the calorific value (in kJ/kg) and *BSFC* is the brake specific fuel consumption (in kg/kWh).

#### CO, NOx, UBHC

Absence of the necessary quantity of oxygen, hastens improper combustion process, which moreover results in identified effluent gases such as unburned hydrocarbons (UBHC), carbon oxide (CO) and oxides of nitrogen (NOx). The AVL DIGAS 444 exhaust gas analyzer was employed to measure the volumes of CO emissions in terms of percentage (%) and UBHC and NOx emissions as ppm.

### Uncertainty analysis

The occurrence of ambiguity during experimentation is probably attributable to a wide variety of causes, some of which may be categorised as instrument error, measurement error, surrounding circumstances, measurement methods, and the kind of instrument. Therefore, in order to determine and provide a feeling of clarity in the measured output answers, each attribute is counted twice for each inquiry run. The error analysis was carried out with the submission of squares for each and every discrete piece of equipment that was measured during the investigation^40^. The incorrect values are detailed in Table 9, which may be found here.

**Table 9:** Errors and uncertainties associated with all instruments.

Measurements	Instrument	Range	Accuracy
Engine load	Strain gauge type load cell	0–25 kg	± 0.1 kg
Speed	Speed sensor	0–10,000 rpm	± 20 rpm
**Calculated results**			**Uncertainty**
Engine power	–	0–50 kW	± 1.0%
Fuel consumption	Level sensor	–	± 1.0%
Air consumption	Turbine flow type	–	± 1.0%
BTE	–	–	± 1.0%
BSEC	–	–	± 1.5%
UBHC	Gas analyzer	Ppm	± 0.1%
CO	Gas analyzer	g/kWh	± 0.2%
NOx	Gas analyzer	Ppm	± 0.1%

The complete proportion of error was valued during the investigation using Eq. (15).

15 $$U= {\left({\left[\frac{\partial R}{\partial {x}_{1}}{W}_{1}\right]}^{2}+{\left[\frac{\partial R}{\partial {x}_{2}}{W}_{2}\right]}^{2}+ \dots \dots . + {\left[\frac{\partial R}{\partial {x}_{n}}{W}_{n}\right]}^{2}\right)}^{1/2}$$

The general percentage of uncertainty (U) = square root of [(Error level in BTE value)^2^ + (Error level in BSEC value)^2^ + (Error level in UBHC value)^2^ + (Error level in CO value)^2^ + (Error level in NO_X_ value)^2^]^1/2^.

The overall percentage uncertainty = Square root of [ (1.0)^2^ + (1.0)^2^ + (1.0)^2^ + (1.5)^2^ + (0.1)^2^ + (1.0)^2^ + (0.2)^2^ + (0.1)^2^]^1/2^.

The general fraction of uncertainty =  ± 2.38%.

The entire uncertainty level throughout experimentation is projected to be ± 2.38%, thus lying-in acceptable range.

## Results and discussion

### Prediction of engine performance and emissions parameters by ANFIS

**Figure 7:**
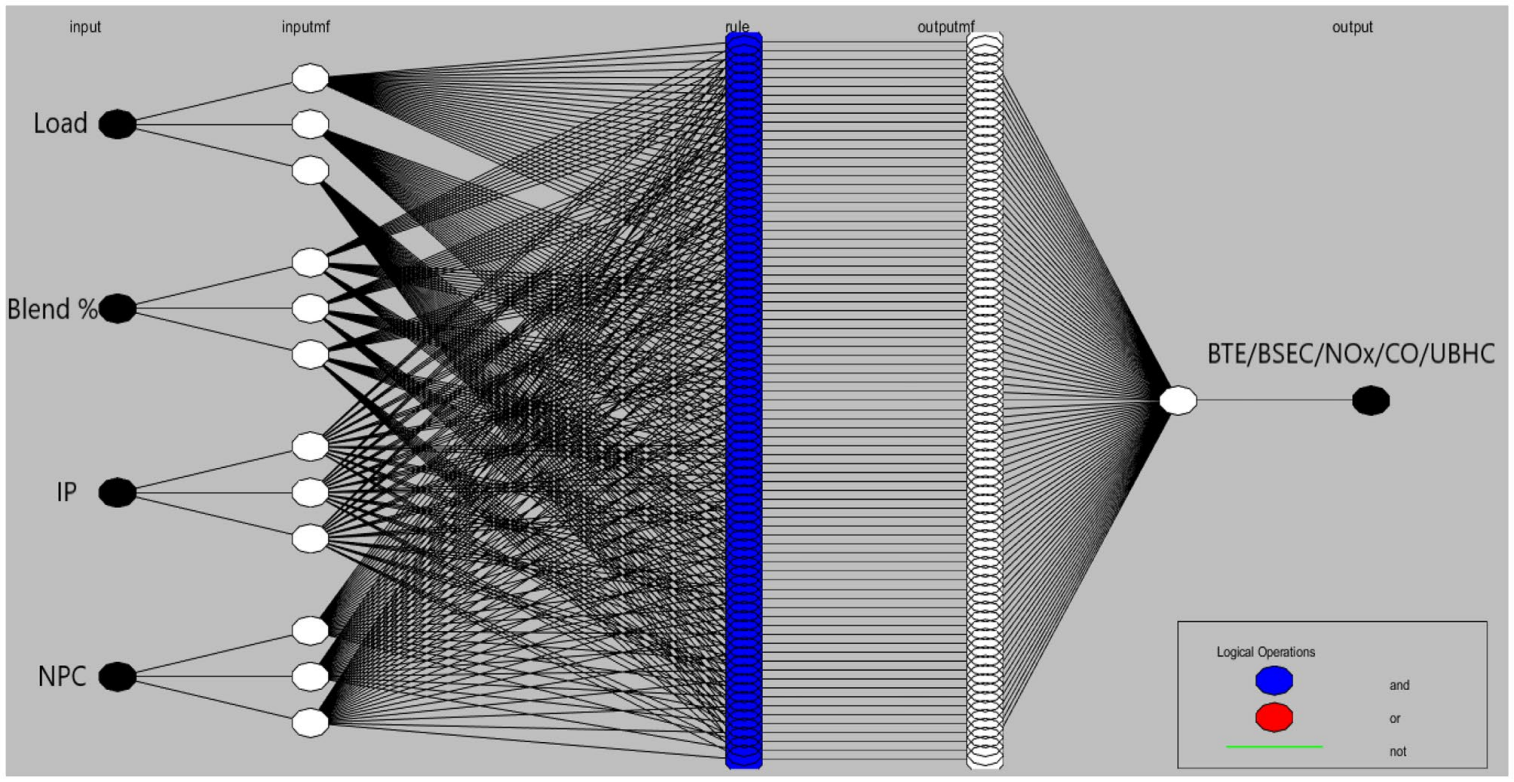
Developed FIS framework with 81 rules for various engine outcomes.

**Figure 8:**
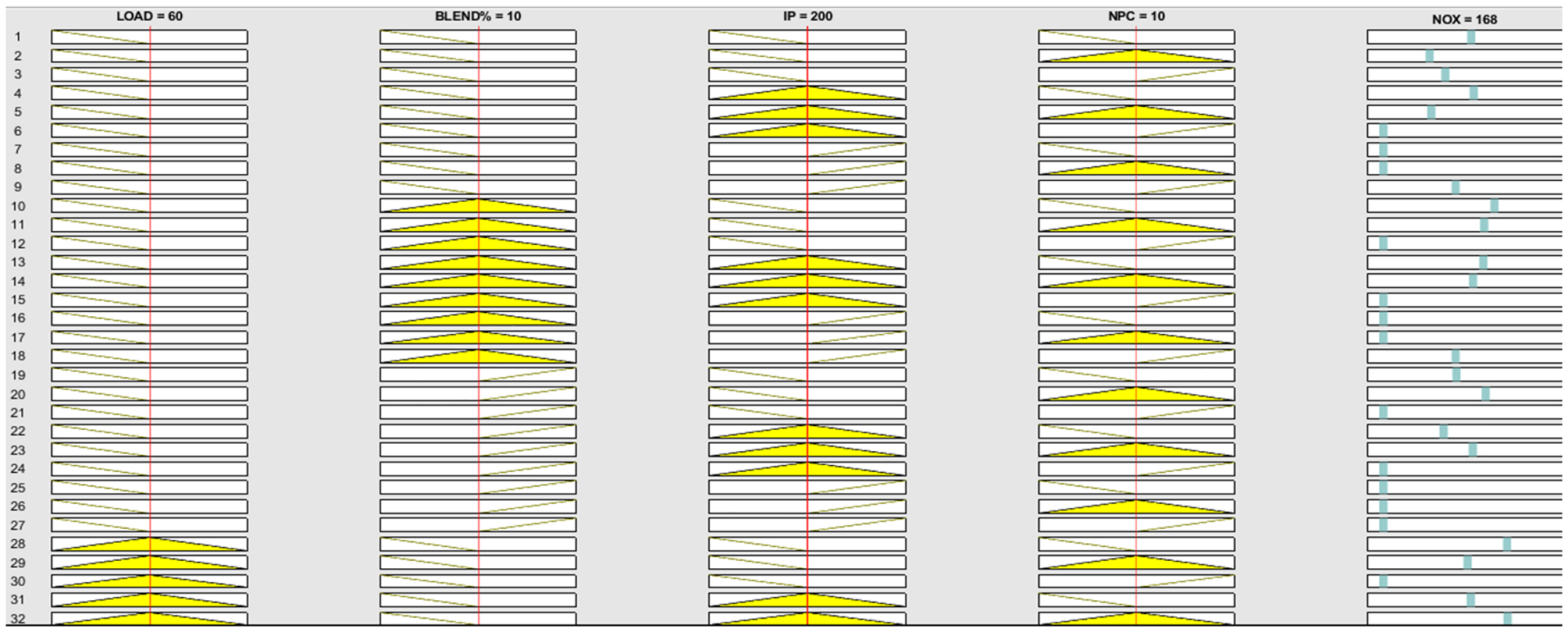
Primary ANFIS data development for a random model (NOx).

**Figure 9:**
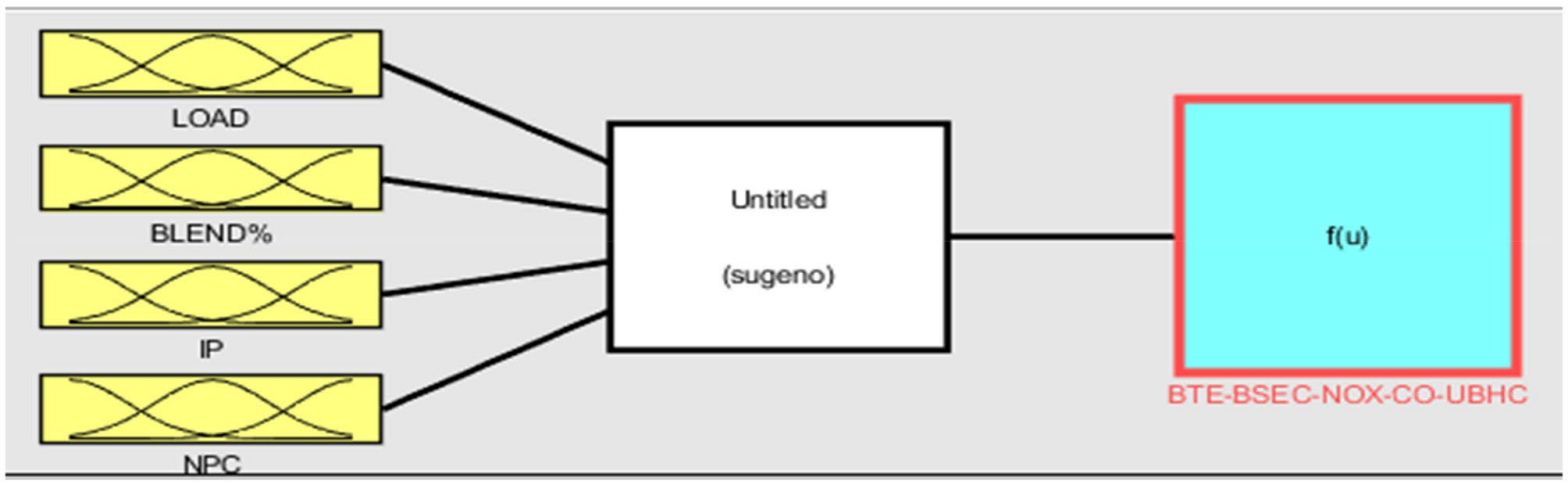
Separate system for each outcome constraints is separately premeditated for set of constarints.

The research employs the ANFIS model which is capable of establishing a feasible relationship between considered inputs and engine performance such as BTE and BSEC and emissions parameters such as UBHC, NOx and CO for both the nanoparticles. Blends of landfill food waste oil was prepared and amalgamated with various nanoparticles in different concentrations. The following input variables combinations might lead to a substantially large data set which consequently furnishes generation of enormous experimental responses eventually consuming time, labour and energy and fuel. The research suggests implementation of a fusion strategy (ANFIS) which facilitates efficient and effective output prediction even for a smaller dataset with negligible errors. The input dataset developed through RSM were trained and validated according to the Sugeno-type fuzzy inference system which primarily works on a complex algorithm employing least square model and the back- propagation gradient descent procedure^37^. To enhance the functionality of the experimental engine characteristics framework, an array-based hybridization was implemented. Previous literature has demonstrated the successful application of the ANFIS framework, which consists of 4 input operators and 5 sheets, in composite manufacturing complications^40^. The fuzzy interface system (FIS) construction for every production parameter was separately intended for the 4 input operators in the ANFIS model, as illustrated in Fig. 7. Within the system, approximately 81 rules were self-developed, a topology that is considered suitable for associating operative parameters and anticipated constraints. Figure 8 provides an example of rules applicable in the AI model for production strictures such as NOx. These proclamations expressed regarding the instructions in AI are inter-related to the Sugeno model, generating its values from predefined datasets obtained from the input responses, as shown in Fig. 9.

**Figure 10:**
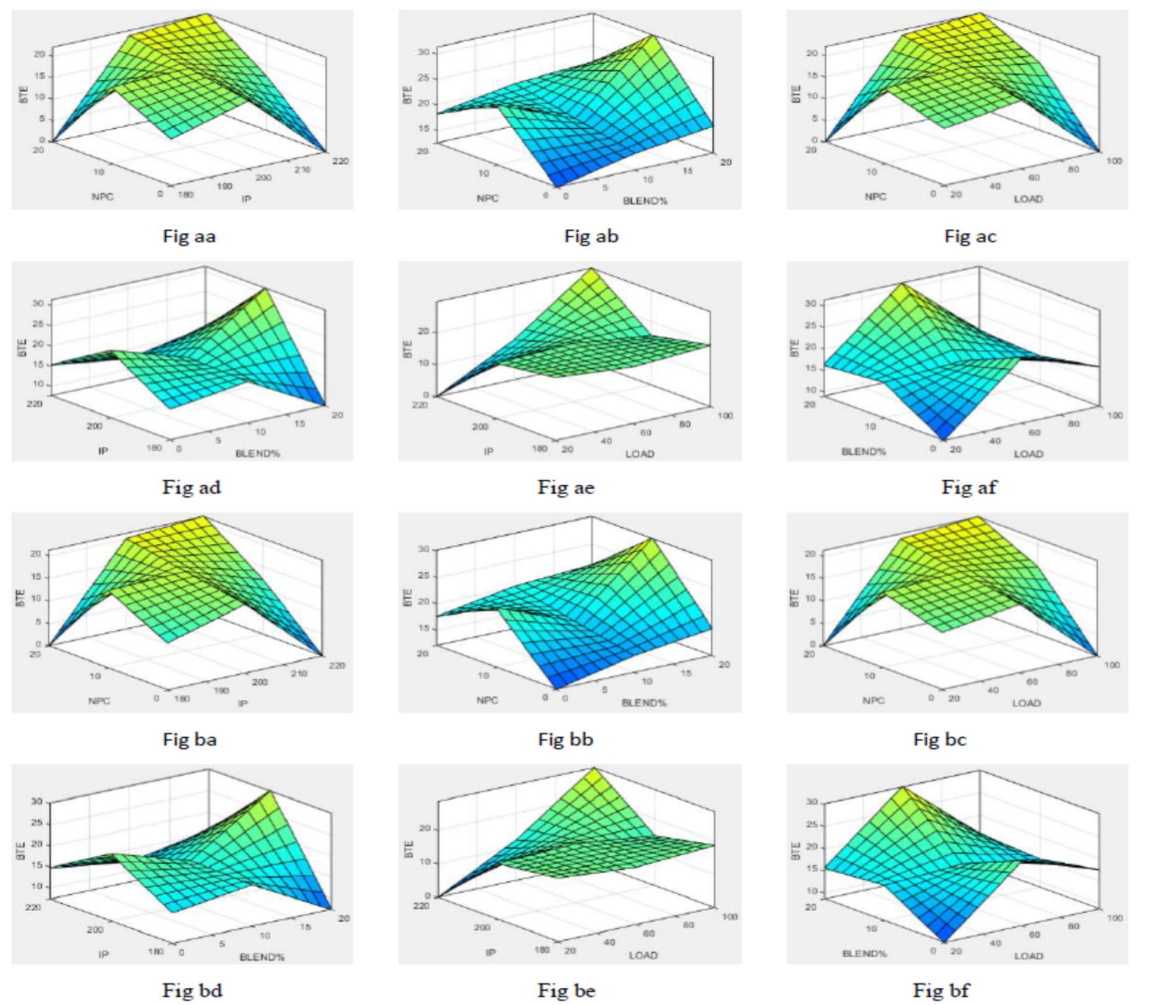
Surface plots for BTE vs Input conditions for ABD (**aa**–**af**) and CBD (**ba**–**bf**).

**Figure 11:**
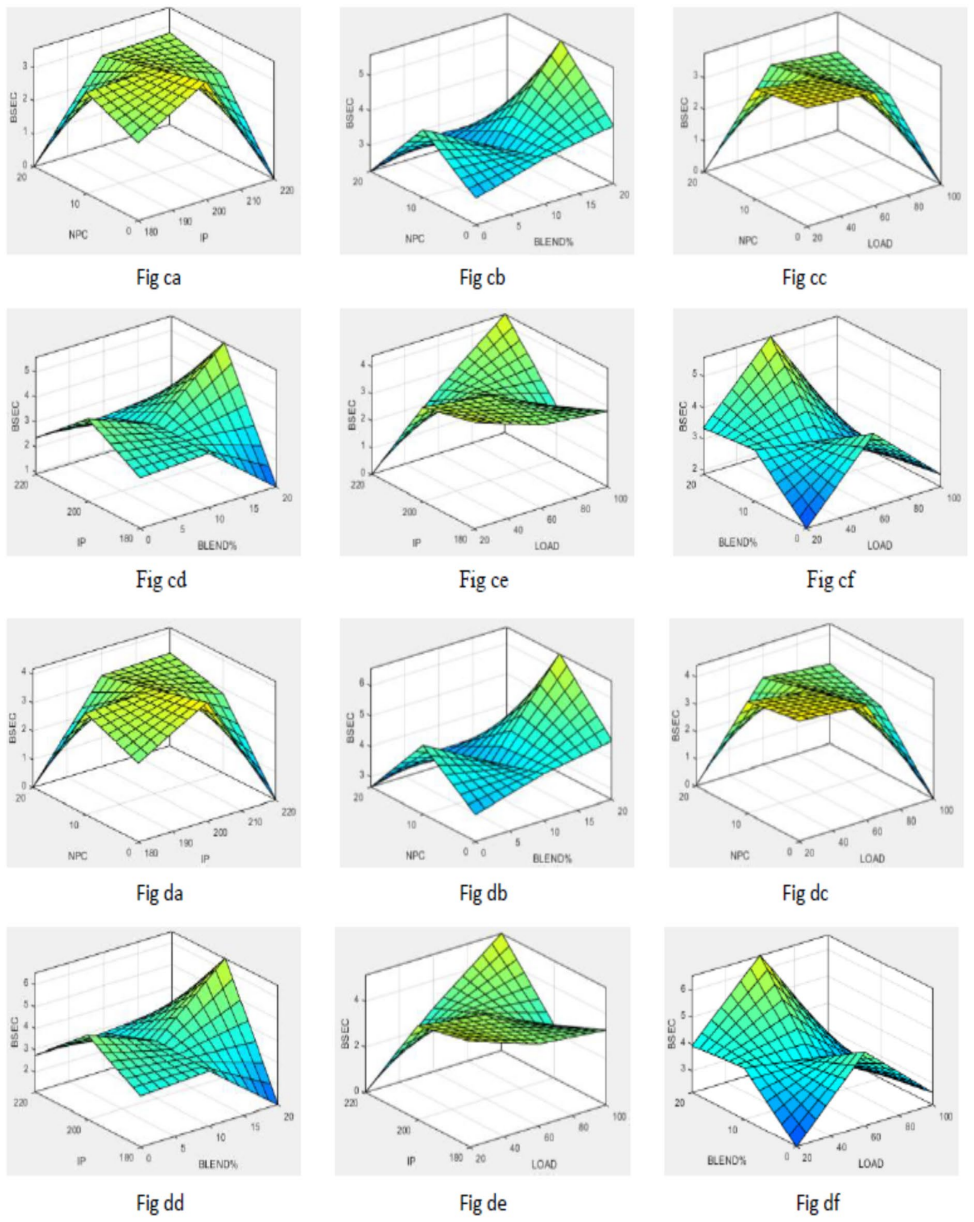
Graphs for BSEC vs constraints ABD (**ca**–**cf**) and CBD (**da**–**df**).

**Figure 12:**
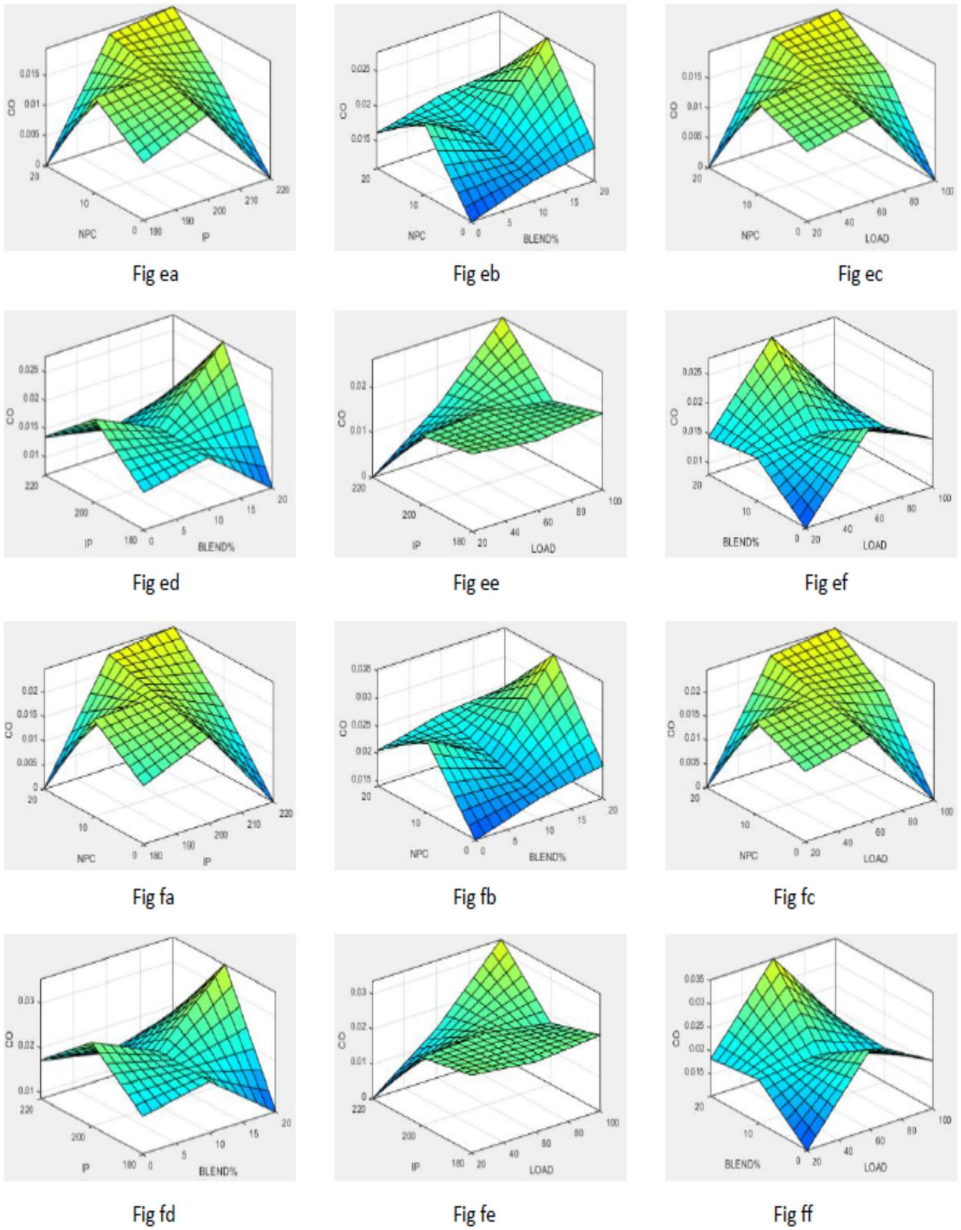
Graphs for CO vs constraints for ABD (**ea**–**ef**) and CBD (**fa**–**ff**).

**Figure 13:**
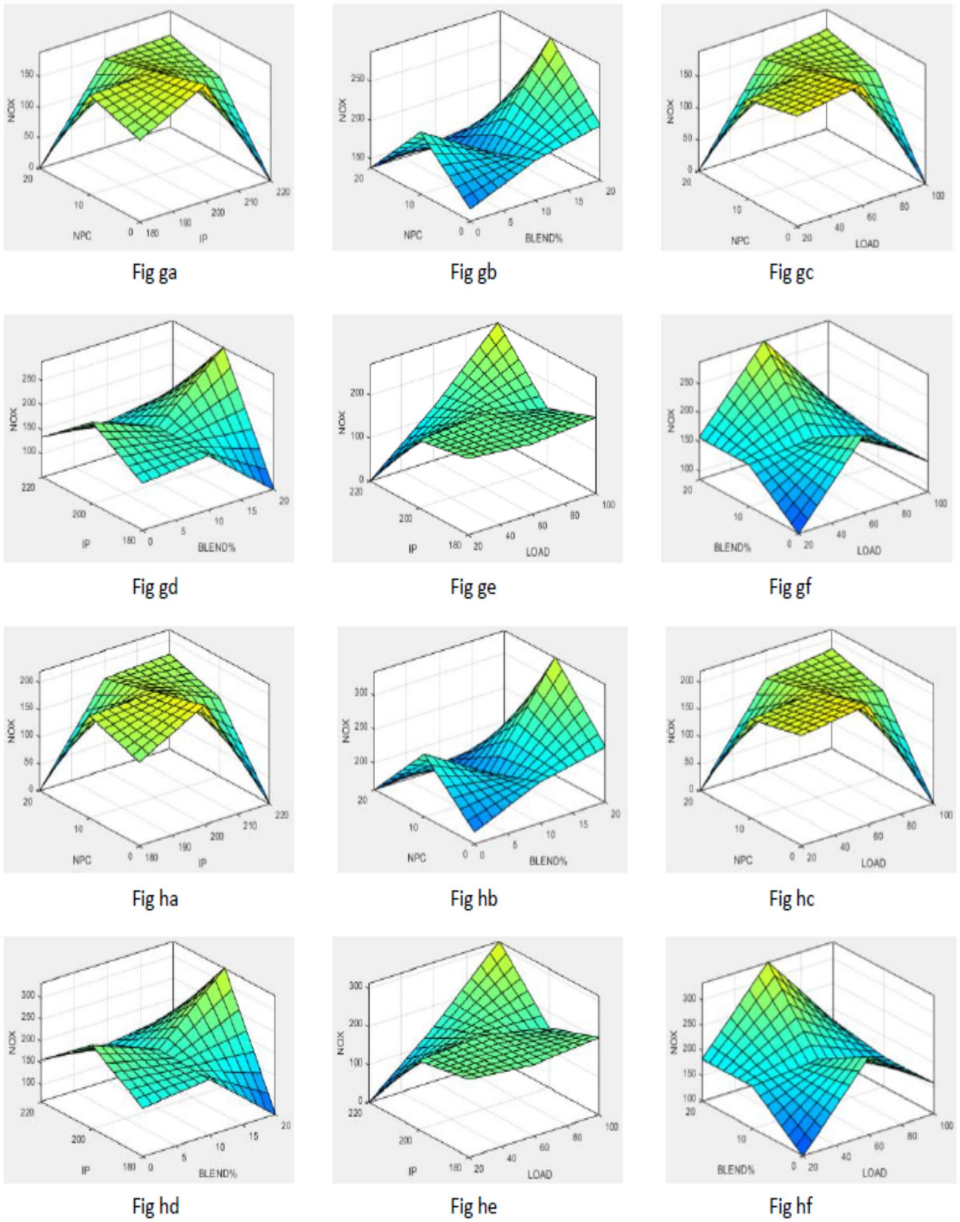
Graphs for NOx vs constraints for ABD (**ga**–**gf**) and CBD (**ha**–**hf**).

**Figure 14:**
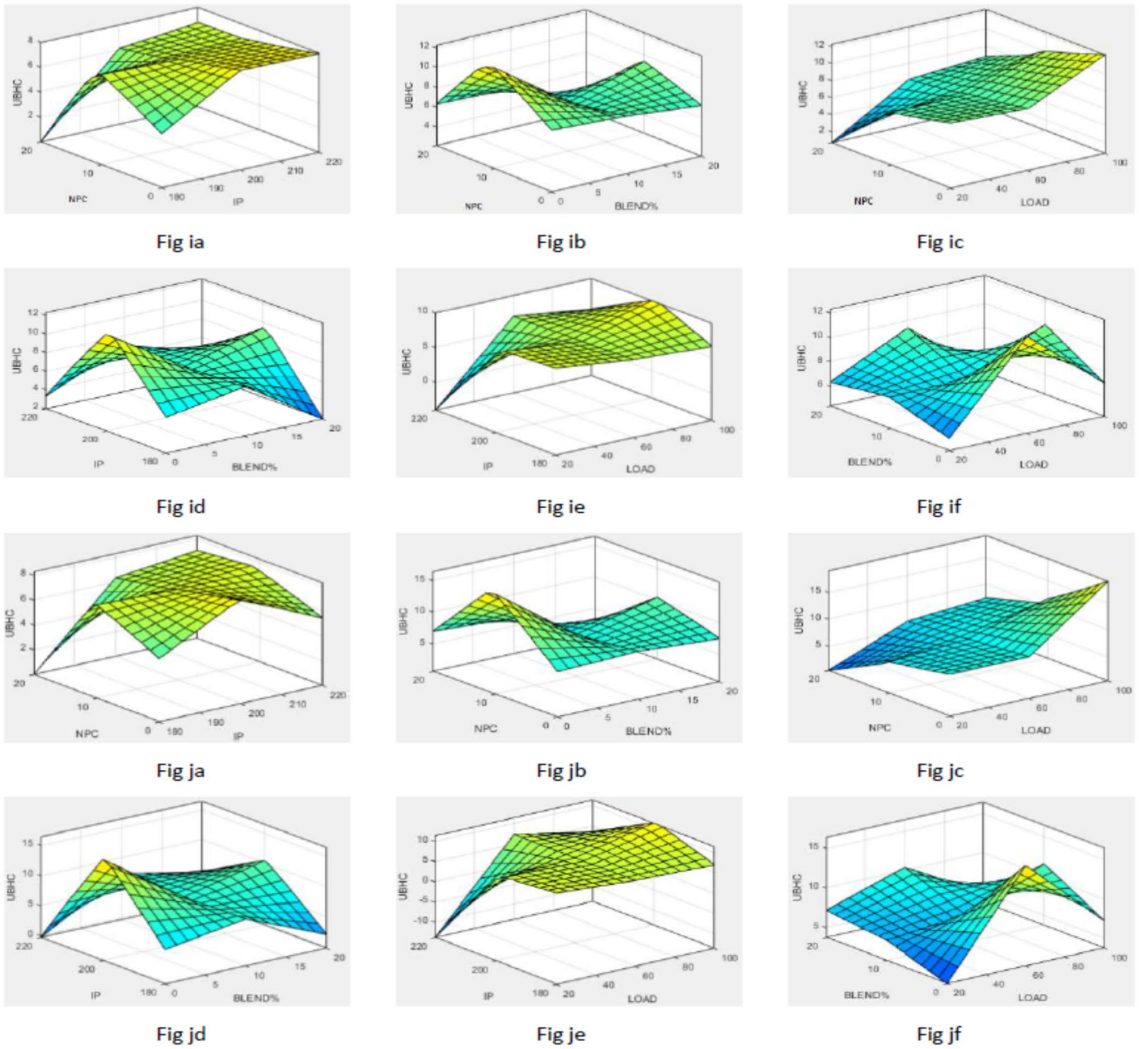
Graphs for UBHC vs Constraints for ABD (**ia**–**if**) and CBD (**ja**–**jf**).

**Figure 15:**
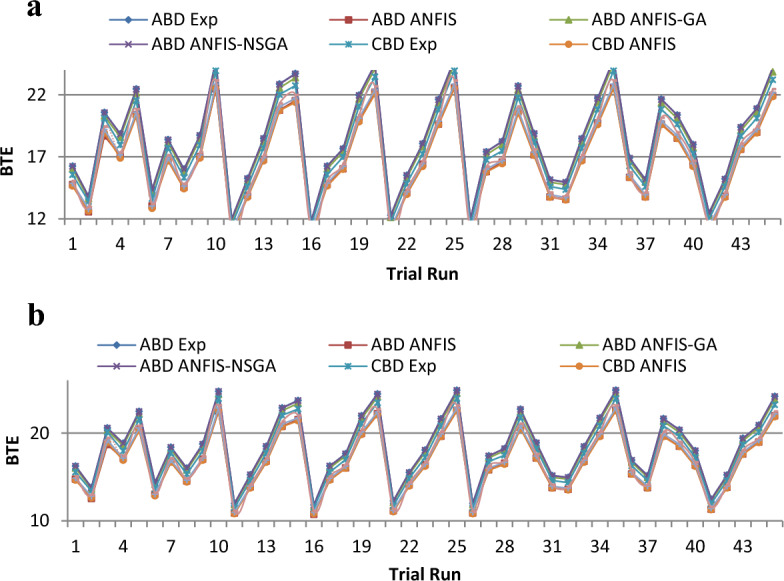
(**a**) Comparative study of observational and expected outcome for BTE Training. (**b**) Comparative study of observational and expected outcome for BTE Testing.

**Figure 16:**
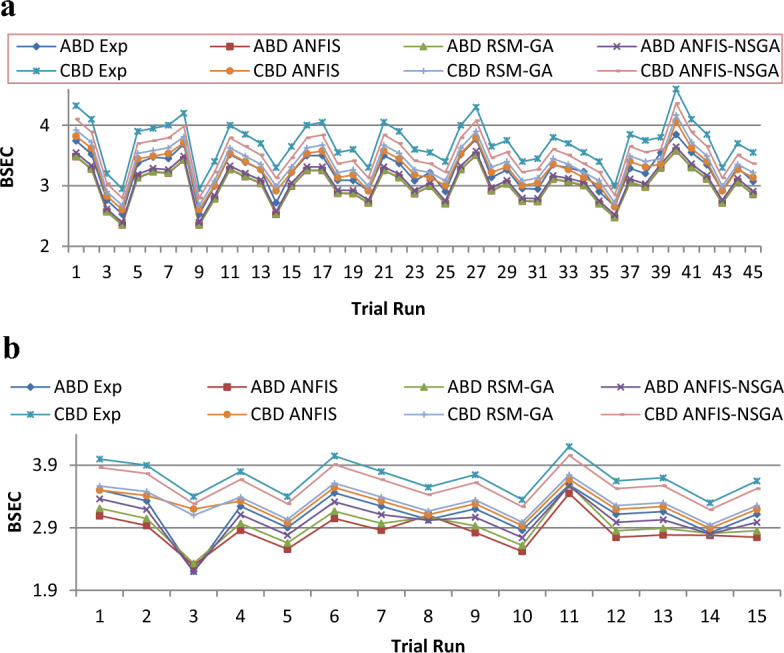
(**a**) Comparative study of observational and expected outcome for BSEC training. (**b**) Comparative study of observational and expected outcome for BSEC testing.

**Figure 17:**
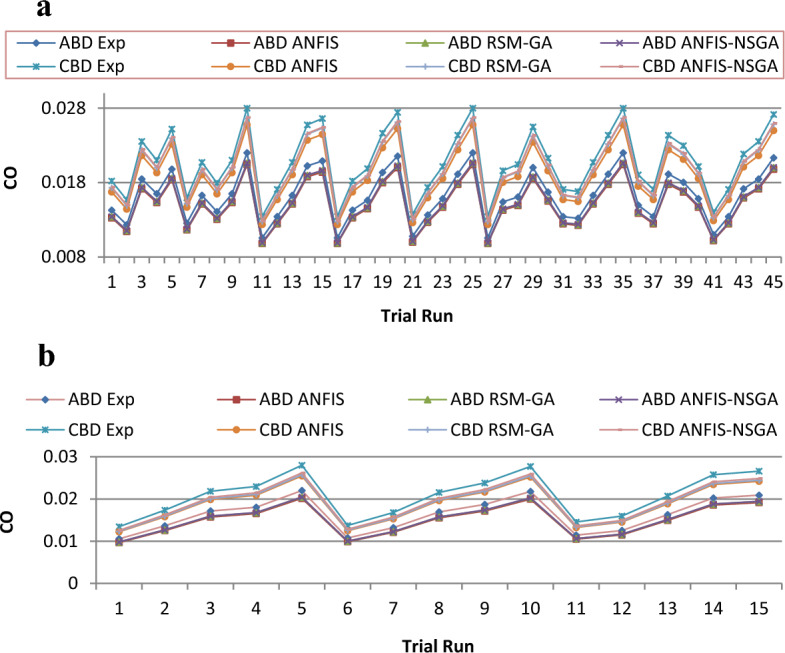
(**a**) Comparative study of observational and expected outcome for CO training. (**b**) Comparative study of observational and expected outcome for CO testing.

**Figure 18:**
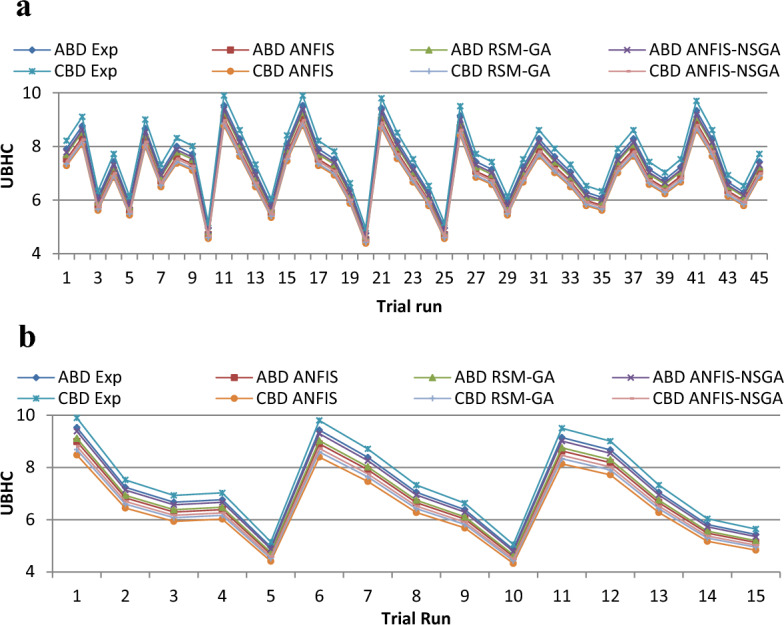
(**a**) Comparative study of observational and expected outcome for UBHC training. (**b**) Comparative study of observational and expected outcome for UBHC testing.

**Figure 19:**
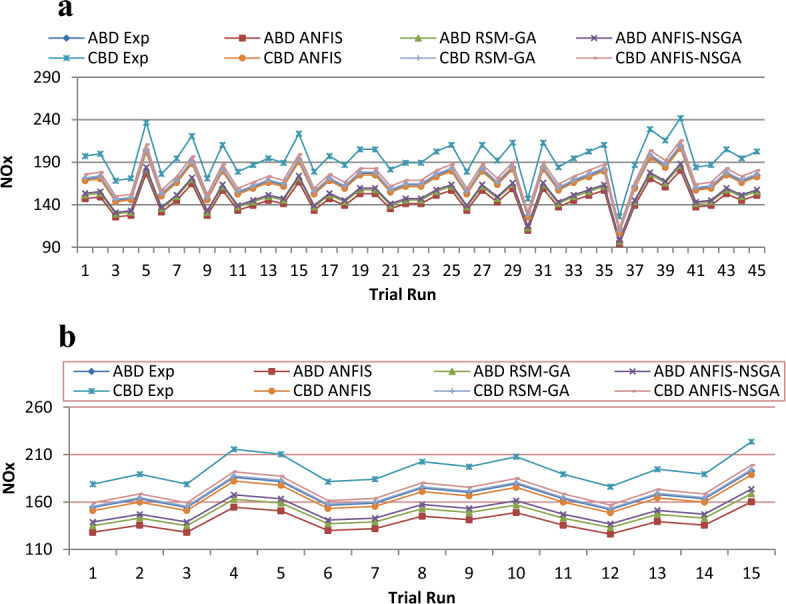
(**a**) Comparative study of observational and expected outcome for NOx Training. (**b**) Comparative study of observational and expected outcome for NOx testing.

To simplify the bend tracing amongst constraints and outcomes for both nano-additives (ABD and CBD), 3-D graphs are established to indicate the inter-relationship amongst any 2 constraints out of four and a one specific outcome, as displayed in various figures. Figure 10 shows plot for BTE versus various input parameters. Figure 11 shows plot for BSEC versus various input parameters. The blend percentage of biodiesel-diesel and the concentration of nanoparticles affect the combustion characteristics and energy release, influencing BSEC. Additionally, the injection pressure plays a crucial role in fuel atomization and distribution, which can alter BSEC. The load percentage directly affects the engine's power requirements and overall efficiency, thus impacting BSEC. Figure 12 shows plot for CO versus various input parameters. The injection pressure plays a critical role in fuel atomization, which can impact the combustion efficiency and subsequently affect CO emissions. Additionally, the load percentage directly influences the engine's power demand and combustion conditions, further affecting CO emissions. Figure 13 shows plot for NOx versus various input parameters nanoparticle concentration affect the combustion process, altering the combustion temperature and oxygen availability, which can lead to variations in NOx formation. Moreover, the load percentage directly influences the engine's power demand and combustion conditions, further affecting NOx emissions. While Fig. 14 is for UBHC as the blend percentage of biodiesel-diesel and nanoparticle concentration affect the fuel–air mixing, which can impact the wholesome combustion process and lead to variations in UBHC emissions. Additionally, the load percentage directly affects the engine's power demand and combustion conditions. Application of ANFIS technique has prognostic efficient outputs identical to those previous experimental generated values employed for training and testing the system for all output responses. The forecasted values for all outcomes, estimated by ANFIS model are graphed in comparison to conventional experimented data in Figs. 15, 16, 17, 18, and 19 respectively to justify systems reliability and correctness.

### Prediction of engine performance and emissions parameters by RSM-GA

Implementing a genetic algorithm in the output answers of the RSM approach allows for additional fine-tuning, which results in better accuracy and efficiency. Estimations and predictions produced using the RSM technique may be used in this way. The use of RSM-GA eradicates the inaccuracies that are caused by the steep descent approach, which causes the results to get trapped inside the local optimum. Utilization of a GA algorithm that randomly searches for solutions enables replies that are both cost-effective and efficient. The genetic algorithm is a kind of adaptive combinatorial search algorithm that is based on the fundamental idea behind biological evolution. This means that the permutations are generated based on the combination of a parent and a kid. The model optimises the fitness functions that are created by the RSM, and as a result, it is able to acquire the most optimal combination of inputs that will result in the best results. In the past, researchers in the field of thermal engineering have confirmed the use of GA by comparing it to other multivariate approaches that take longer to provide results^31,32^. Therefore, using a GA weighted sorting approach in RSM derived predictions reduces the uncertainty in membership functions, which makes improvisation easier.

A fitness function value was generated and defined in the workspace of the software. The development of the fitness equation involved considering the mean statistical error values for all datasets between the predicted and experimental responses. The fitness function was recalled when inputting values into the GA toolbox. The combination of soft computing techniques with an optimization model (GA) provides a faster and more efficient architecture procedure, resulting in the best of both systems. Comparative results are displayed in Figs. 15, 16, 17, 18, and 19. To validate the fitting function, R^2^ values were estimated to be between 0.85 and 1 for all outcomes, suggesting accurate fitting of data in the RSM-GA model. Henceforth, RSM-GA algorithm is stipulated to be a powerful tool for modelling the engine performance and emission characteristics.

### Prediction of engine performance and emissions parameters by ANFIS-NSGA-II

Initially input data conditions are uploaded in the FIS model, which generates separate outcomes individually for each model. In particular, current setup is again reoptimized with the help of using capable NSGA-II procedure which will establish the outcomes of the study in a much more clear way. The constraints of the study conform to global ethics for petro-diesel engine operation. The advanced system of NSGA-II system can be excellently functional in multiple domains for variable-functional convergence procedures, pertaining to nearer approximations^40,41^. Moreover, adjusted populace is organized more on source of in dominated pattern, eventually leading to development of frontages. The primary front was entirely non-dominant set in the existing population whereas the secondary front was dominated by the entities in the primary front only and so on. Soft computing techniques like genetic algorithms and its hybrid versions are often utilized for multi-objective optimization in diesel engines. These optimization processes involve complex non-linear equations with contrasting objectives. The Pareto optimal front set is often utilized to obtain the best operating conditions for the desired engine outputs. In this regard, the elitist NSGA-II structure has been successfully applied in several fields to facilitate nearer conjunction with suitable categorization built on precedence.

The ANFIS-NSGA-II model is a hybrid model that combines the advantages of both soft computing techniques and optimization models. It was successful in forecasting exact outcomes within advanced oversimplification ability for performance-emission parameters for petro-diesel machines. This model provides a Pareto optimal front set that represents the finest counter and validation among the considered objective functions predefined within the system. The improved outcomes extracted with the aid of above procedure can be explained through values presented Table 10, where ideal load application for engine can be 100%, ideal mix ratio can be in following ratio which is 20%, ideal nano-additive requirement in the blend is close to 20 ppm, while ideal pressure inserted within the cylinder can be 200 bar. These conditions yield BTE to be 24.45 kW, BSEC to be 2.761784 NOx to be 159.5488 ppm, UBHC to be 4.687807 ppm, and CO to be 0.020243%. A favourite prioritization is established in case of the attained outputs of the study where BTE is assigned with zenith while others are assumed with utmost similarly.

**Table 10:** Pareto optimal front set.

Trial run	Load	Blend %	IP	NPC	BTE	BSEC	NOx	UBHC	CO	
26	100	20	220	20	24.86	2.74	163.63	4.87	0.020	
49	60	10	190	15	13.35	2.11	138.93	6.57	0.015	
37	20	0	180	20	16.89	2.51	98.18	7.50	0.014	
21	100	20	200	20	24.43	2.76	159.54	4.68	0.020	
47	20	0	180	0	11.11	3.36	138.93	9.38	0.010	

Moreover, the developed fitness equation was processed by taking into consideration mean numerical fault standards for above datasets amongst the investigational and prognostic outputs. While alimentation data’s are fed into the GA, the appropriateness function was recalled. This hybrid model provides the finest of mutually organizations, permitting a sooner and well-organized planning procedure. Additionally, the ANFIS-NSGA-II model was compared with other models such as ANFIS and RSM-GA, and it aligned itself closest to the generated experimental values, furnishing superiority to the other models for data prediction. Thus, the overhead declaration rationalizes and confirms the appropriateness of the perto-diesel machine characteristics analysed by commissioning the ANFIS-NSGA system to investigate and augment variables.

### Comparative study of the predicted values of the developed models

**Figure 20:**
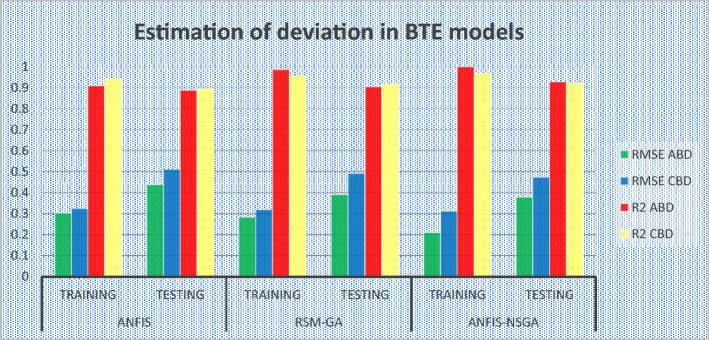
Comparative study of various BTE models.

**Figure 21:**
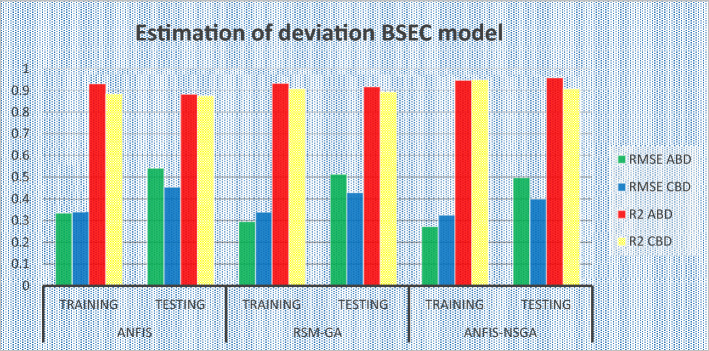
Comparative study of various BSEC models.

**Figure 22:**
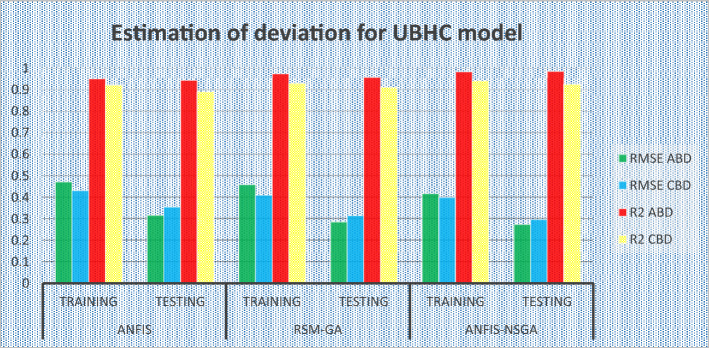
Comparative study of various UBHC models.

**Figure 23:**
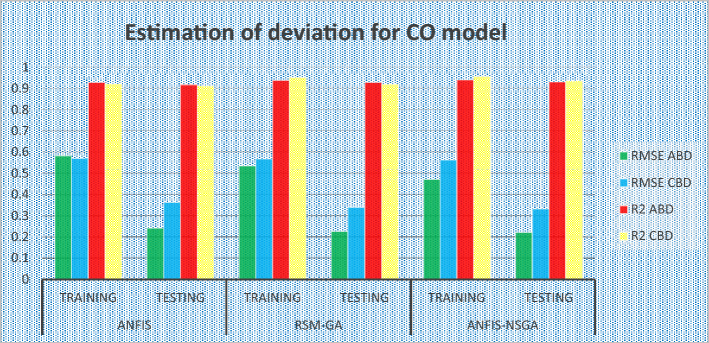
Comparative study of various CO models.

**Figure 24:**
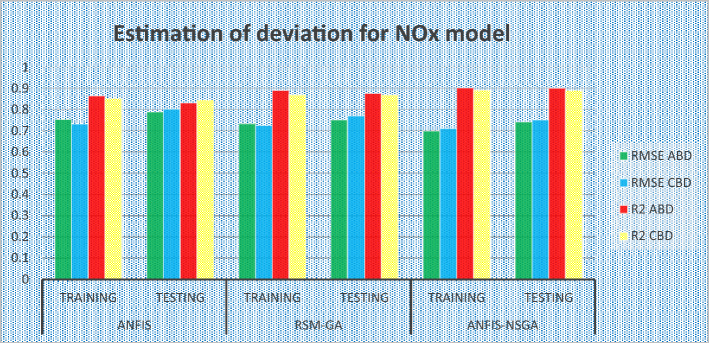
Comparative study of various NOx models.

The outcomes predicted by hybrid models (ANFIS, RSM-GA and ANFIS-NSGA-II) were evaluated on the basis of regression formulas such as root mean square error (RMSE) and fraction of variance R^2^. Often these statistical tools are employed to estimate the deviation between the experimental and predicted responses. Fraction of variance (R^2^) works on the concept of linear regression taking in account all the over and under estimations within the system. RMSE is employed to estimate how close the residuals (experimental data and forecasted data) are to the best fit line. To validate and cross verify the engine outcomes such as BTE, BSEC, UBHC, CO and NOx, these were tested for different uncertainties using statistical tools. Different types of regression analysis were performed to evaluate the feasibility of these soft computed hybrid models developed for engine testing. The accuracy of the predictive model was validated by considering the regression formulas like RMSE, and R^2^. Tables 11, 12, 13, 14 and 15 presents a comparative analysis of the regression errors evaluated for the predicted outcomes such as BTE, BSEC, UBHC, CO and NO_X_ respectively. Also, the graphs Figs. 20, 21, 22, 23 and 24 show comparative variations and deviations among different models. Previous prediction models were deemed accurate if RMSE evaluated was close to zero. Conversely, the fraction of variance of the forecasted data should be close to 1 for accurate fitting model. In the present research, all implemented models complied with the above statistical error standards facilitating a reliable and consistent forecast. The ANFIS-NSGA-II model displayed significantly improved accuracy in predicting output responses when compared to ANFIS and RSM-GA models. The error values generated by the ANFIS-NSGA-II model were very close to the experimental responses. In addition, the R^2^ training values for ANFIS-NSGA-II model (ABD blend) for BTE, BSEC, UBHC, CO and NOx (0.9985, 0.9464, 0.9838, 0.9389 and 0.9011) were superior to RSM-GA (0.9856, 0.9318, 0.9739, 0.9372 and 0.8901) and ANFIS (0.9075, 0.9294, 0.9513, 0.9281 and 0.8641) models. This indicates that the ANFIS-NSGA-II framework is more reliable and accurate in developing fuzzy relationships as compared to its counterparts. The plotted graphs confirm that the soft computing techniques have accurately predicted the values which are close to the experimental values. The integration of ANFIS-NSGA-II model yielded better results in comparison to ANFIS and RSM-GA models.

**Table 11:** Comparative study of various BTE models.

		ANFIS		RSM-GA		ANFIS-NSGA	
		Training	Testing	Training	Testing	Training	Testing
ABD	RMSE	0.30287	0.438	0.2838	0.3915	0.2105	0.3793
	R^2^	0.9075	0.8872	0.9856	0.9037	0.9985	0.9273
CBD	RMSE	0.325	0.511	0.320	0.491	0.313	0.473
	R^2^	0.941	0.893	0.955	0.915	0.967	0.921

**Table 12:** Comparative study of various BSEC models.

		ANFIS		RSM-GA		ANFIS-NSGA	
		Training	Testing	Training	Testing	Training	Testing
ABD	RMSE	0.3349	0.541	0.2951	0.5121	0.2721	0.4971
	R^2^	0.9294	0.8815	0.9318	0.9151	0.9464	0.9584
CBD	RMSE	0.3411	0.4544	0.339	0.4293	0.3252	0.3999
	R^2^	0.8834	0.8756	0.9067	0.8921	0.9481	0.9061

**Table 13:** Comparative study of various UBHC models.

		ANFIS		RSM-GA		ANFIS-NSGA	
		Training	Testing	Training	Testing	Training	Testing
ABD	RMSE	0.4715	0.315	0.4578	0.2848	0.4153	0.2726
	R^2^	0.9513	0.9437	0.9739	0.9569	0.9838	0.9851
CBD	RMSE	0.4309	0.3534	0.4080	0.3133	0.3981	0.2954
	R^2^	0.921	0.890	0.929	0.911	0.9415	0.925

**Table 14:** Comparative study of various CO models.

		ANFIS		RSM-GA		ANFIS-NSGA	
		Training	Testing	Training	Testing	Training	Testing
ABD	RMSE	0.5825	0.2411	0.5342	0.2251	0.4703	0.2204
	R^2^	0.9281	0.9171	0.9372	0.9283	0.9389	0.9303
CBD	RMSE	0.5706	0.3621	0.5666	0.3397	0.5606	0.3320
	R^2^	0.9201	0.910	0.9510	0.919	0.9555	0.935

**Table 15:** Comparative study of various NOx models.

		ANFIS		RSM-GA		ANFIS-NSGA	
		Training	Testing	Training	Testing	Training	Testing
ABD	RMSE	0.7531	0.7877	0.7328	0.7515	0.6981	0.7407
	R^2^	0.8641	0.8301	0.8901	0.8751	0.9011	0.9001
CBD	RMSE	0.7309	0.801	0.7245	0.7696	0.7099	0.7511
	R^2^	0.8525	0.8441	0.8695	0.8691	0.8909	0.8899

### Discussion

Four input parameters were chosen: Blend percentage (B), nano-particle concentration (NPC), engine load (LD), and ignition pressure (IP). The feasibility of these parameters was confirmed through experimental results from previous research. The researchers considered the feasible range of each parameter that strongly influenced the output responses. For Blend percentage, the range was set between 5 and 20%, as beyond 20% would result in high viscosity and low density, requiring modifications to the original engine design and making the process infeasible and expensive. Similarly, NPC was limited to 20 ppm, as exceeding this concentration would lead to excessive deposition and increased aggregation of nano-additives, causing segregation among fuel blends. Regarding Load, levels below 20% were not considered as they presented small negligible variations in engine performance and exhaust analysis, and thus were not deemed influential enough to be included in the study. Finally, IP was restricted to the range of 180–220 bars, as values above 220 bars would lead to abnormal engine temperatures, potentially resulting in engine seizure or malfunction.

In the quantitative analysis, the researchers utilized statistical and mathematical methods to analyze the relationship between the selected input parameters and the output responses. They conducted numerous experimental runs by varying the levels of blend percentage (B), nano-particle concentration (NPC), engine load (LD), and ignition pressure (IP) as per the chosen discrete levels within their feasible ranges. The performance-emission characteristics of the biodiesel engine were recorded for each experimental run, generating a comprehensive dataset. Statistical techniques such as regression analysis, analysis of variance (ANOVA), and correlation analysis were employed to identify the significant effects of individual parameters and their interactions on the engine outputs. This quantitative analysis provided valuable insights into the quantitative contributions of each input parameter to the performance and emission characteristics of the biodiesel engine, allowing for a deeper understanding of their influence and potential optimization strategies.

In the qualitative analysis, the researchers focused on understanding the practical implications of the selected input parameters in real-world engine applications. They considered the physical limitations and engineering constraints associated with the chosen input parameter ranges. For instance, the decision to limit the biodiesel blend percentage (B) to 20% was based on the consideration that higher concentrations would lead to unfavorable engine properties, making it impractical for conventional engine designs. Similarly, the restriction on nano-particle concentration (NPC) to 20 ppm was due to potential complications in the engine's valve timing diagram and particle deposition issues. By qualitatively analyzing the effects of each parameter, the researchers ensured that the experimental conditions were not only scientifically relevant but also practically achievable in diesel engine applications. This qualitative assessment provided valuable insights into the feasibility of implementing the findings in real-world scenarios and helped avoid potential engineering challenges or performance issues.

## Validation of proposed model with previous literature

In the part before this one, the intelligent model ANFIS-NSGA showed a lower error rate (RSME) in contrast to the single ANFIS model and the RSM-GA model. In addition to this, a zenith R^2^ value that was close to 1 was accomplished while using the exact same integrated model. Previous models were recollected and compared with the current hybrid model, which is provided in Table 16. This was done in order to validate and justify the selection of the ANFIS-GA model. On the basis of the results of a statistical survey, a comparison of the data for the various engine-based outcomes was developed. The ANFIS-NSGA-II model has exhibited a similar trend of improved precision, characterized by reduced RSME and increased R^2^, as seen in previous studies. This outcome substantiates the effectiveness of the proposed fuzzy-optimization integrated framework and aligns with previous findings that have also demonstrated a similar pattern of higher accuracy.

**Table 16:** Comparison of prediction capability of various models and developed ANFIS-NSGA-II.

References	Model	Fuel	RMSE	R^2^
Seraj et al.^24^	ANFIS-GA	Eucalyptus	3.470	0.38
Khan^42^	ANFIS	Eichhornia Crassipes	6.426	0.24
Aghbashlo et al.^43^	ANFIS-ALFIMO	Waste cooking oil	0.423	0.92
BTE (current study) (ABD)	ANFIS-NSGA-II	Waste food oil	0.210	0.99
BSEC (current study) (ABD)	ANFIS-NSGA-II	Waste food oil	0.272	0.93
NOx (current study) (ABD)	ANFIS-NSGA-II	Waste food oil	0.698	0.90
CO (current study) (ABD)	ANFIS-NSGA-II	Waste food oil	0.470	0.93
UBHC (current study) (ABD)	ANFIS-NSGA-II	Waste food oil	0.415	0.94

## Conclusion, limitations and future scope

### Conclusions

In conclusion, the study employed a comprehensive approach, utilizing both quantitative and qualitative analyses, to investigate the influence of selected input parameters on the performance and exhaust characteristics of biodiesel engines. Through quantitative analysis, the researchers analyzed a rich dataset obtained from experimental runs, employing statistical techniques such as regression analysis, ANOVA, and correlation analysis. This enabled the identification of significant effects and interactions among the input parameters, providing valuable quantitative insights into their contributions to engine outputs. The results of the quantitative analysis shed light on the optimization potential of input parameters allowing for informed decision-making in engine design and operation.

Simultaneously, the qualitative analysis considered practical aspects and engineering constraints related to the selected input parameter ranges. By evaluating the feasibility of applying the chosen parameters in real-world engine applications, the researchers ensured that their findings were not only scientifically sound but also practically viable. Limiting the biodiesel blend percentage (B) to 20% due to viscosity and density concerns, capping the nano-particle concentration (NPC) at 20 ppm to avoid valve timing and particle deposition issues, and restricting the engine load (LD) below 20% to account for negligible variations in engine performance exemplify the qualitative assessment's importance. The qualitative analysis provided valuable insights into the practicability of implementing the study's findings in practical diesel engine setups, helping prevent potential engineering challenges and performance limitations.

The contemporary research explored the potential of landfill food waste oils for various input conditions such as blend percentage (BP), load (LD), ignition pressure (IP) and nanoparticle concentration (NPC). Also, two types of nanoparticles namely aluminium oxide and copper oxide were employed so as to predict the best engine characteristics obtained among them. The engine experimental responses such as BTE, BSEC, UBHC, CO and NOx were generated and compared with those obtained by hybrid soft computing techniques. In addition, the optimization techniques provided the optimal combination of engine inputs, which resulted in the best possible conditions for the utilization of landfill waste biodiesel fuel in a diesel engine. Here are some of the most important results of the study:

- The Landfill waste (leachate) oils have been established as a novel feedstock after qualitative research.
- Intelligent computational system is developed by integrating the attributes of ANFIS prediction and GA optimization (GA and NSGA-II) to prepare a comparative analysis between generated outcomes and experimental values for diesel engine characteristics.
- Statistical tools such as RSME and R^2^ were employed to estimate the error rate.
- ANFIS-NSGA-II hybrid model forecasted outcomes with better efficiency since MSE and RSME values were lower in comparison to conventional ANFIS model.
- Qualitative research has been prepared to develop hybrid models which can predict engine characteristics with minimum experimentation dataset, quickly and efficiently.
- Outcomes generated by ANFIS-NSGA-II were more precise and efficient in comparison to other models.
- Optimum results were achieved after employing multi-objective function optimisation (ANFIS-NSGA-II) for BTE, BSEC, NOx, UBHC and CO which were 24.45 kW, 2.76, 159.54 ppm, 4.68 ppm, and 0.021%.

### Limitations

All researches are bound to have some constraints or flaws in the methodology research design, technique, materials, etc., and these factors may impact the findings of your study. It is necessary to acknowledge any limitations in the research paper in order to aware the readers of the potential shortcomings which might affect the conclusions drawn from the research. Like other studies, following are the limitations of the present study:

- Biofuel amalgamation was only explored for metallic nanoparticles which somewhat restricts the comparisons.
- Fewer number of operating conditions (four) were considered for conducting experimental investigation which might not be sufficient for a variety of engines such as aeroplane and ship engines.
- Combustion analysis could draw out more comparisons between the two nanofluids.
- Limited number of outcomes were explored.

### Scope for future work

The boundaries or restrictions of the research explores more domains directing forthcoming studies and guide to future examiners to strategize and accomplish experimental work in diesel engine. Subsequent points explain possible prospects for future investigators to go through:

- A comparative analysis between several nanoparticles (of both nature metallic and non-metallic) needs to be carried out and ranked from best to worst with the aid of multi-criteria decision methods for diesel engine performance and emission.
- A broader experimental dataset might be prepared in future having higher number of input parameters.
- More output responses including other combustion parameters might be measured and included for better comparison in future.

## Data Availability

The data supporting the findings of this study are available from the corresponding author upon reasonable request.

## Abbreviations

BSEC: Brake specific energy consumption
B0: 0% Blending (landfill waste biodiesel) with diesel
B5: 5% Blending (landfill waste biodiesel) with diesel
UBHC: Unburnt hydrocarbons
B10: 10% Blending (landfill waste biodiesel) with diesel
B15: 15% Blending (landfill waste biodiesel) with diesel
CO: Carbon monoxide
B20: 20% Blending (landfill waste biodiesel) with diesel
LFB: Landfill waste biodiesel
RSM: Response surface methodology
ANFIS: Adaptive neuro-fuzzy inference system
ANN: Artificial neural network
GA: Genetic algorithm
NOx: Oxides of nitrogen
NSGA: Non-sorting genetic algorithm
ABD: Aluminium oxide biodiesel
CBD: Copper oxide biodiesel
BTE: Brake thermal efficiency
FFA: Free fatty acids
